# Prognostic impact of circulating tumor *RAS* and *BRAF* mutation dynamics in patients with metastatic colorectal cancer

**DOI:** 10.1016/j.isci.2026.117112

**Published:** 2026-08-13

**Authors:** Hiroki Osumi, Eiji Shinozaki, Yoshiaki Nakamura, Taito Esaki, Hisateru Yasui, Hiroya Taniguchi, Hironaga Satake, Yu Sunakawa, Yoshito Komatsu, Yoshinori Kagawa, Tadamichi Denda, Manabu Shiozawa, Taroh Satoh, Tomohiro Nishina, Toshifumi Yamaguchi, Naoki Takahashi, Takeshi Kato, Hideaki Bando, Kensei Yamaguchi, Takayuki Yoshino

**Affiliations:** 1 Department of Gastroenterology, Kanagawa Cancer Center, Yokohama, Japan; 2 Department of Gastroenterological Chemotherapy, Cancer Institute Hospital of Japanese Foundation for Cancer Research, Tokyo, Japan; 3 Department of Gastroenterology and Gastrointestinal Oncology, National Cancer Center Hospital East, Kashiwa, Japan; 4 Department of Gastrointestinal and Medical Oncology, National Hospital Organization Kyushu Cancer Center, Fukuoka, Japan; 5 Department of Medical Oncology, Kobe City Medical Center General Hospital, Kobe, Japan; 6 Department of Clinical Oncology, Aichi Cancer Center Hospital, Nagoya, Japan; 7 Department of Medical Oncology, Kochi Medical School, Kochi, Japan; 8 Department of Clinical Oncology, St. Marianna University School of Medicine, Kawasaki, Japan; 9 Department of Cancer Chemotherapy, Hokkaido University Hospital Cancer Center, Sapporo, Japan; 10 Department of Gastroenterological Surgery, Osaka International Cancer Institute, Osaka, Japan; 11 Division of Gastroenterology, Chiba Cancer Center, Chiba, Japan; 12 Department of Gastrointestinal Surgery, Kanagawa Cancer Center, Yokohama, Japan; 13 Palliative and Supportive Care Center, Osaka University Hospital, Suita, Japan; 14 Department of Gastrointestinal Medical Oncology, National Hospital Organization Shikoku Cancer Center, Ehime, Japan; 15 Cancer Chemotherapy Center, Osaka Medical and Pharmaceutical University Hospital, Osaka, Japan; 16 Department of Gastroenterology, Saitama Cancer Center, Saitama, Japan; 17 Department of Surgery, NHO Osaka National Hospital, Osaka, Japan

**Keywords:** circulating tumor DNA, metastatic colorectal cancer, NeoRAS, NeoBRAF, overall survival

## Abstract

Post-treatment alterations in circulating tumor DNA (ctDNA) hold potential for refining treatment strategies in metastatic colorectal cancer (mCRC). In this multi-institutional study of 1,391 patients from the SCRUM-Japan GOZILA platform, we evaluated the prognostic significance of post-treatment rat sarcoma viral oncogene homolog (*RAS*) and v-raf murine sarcoma viral oncogene homolog B1 (*BRAF*) V600E mutation (MT) dynamics by comparing baseline tissue profiling with subsequent longitudinal ctDNA analysis. While patients with Neo*RAS* or Neo*BRAF* wild-type (WT) achieved an overall survival (OS) comparable to the persistent *RAS* WT cohort, those with persistent or acquired mutations demonstrated significantly impaired outcomes. Multivariable analysis identified baseline tissue *RAS* and *BRAF* MT status before treatment initiation, Neo*BRAF* WT, ctDNA fraction, number of treatment lines at the time of sampling, and prior anti-vascular endothelial growth factor therapy as independent correlates of OS. These results show that post-treatment circulating tumor *RAS* and *BRAF* MT dynamics may serve as useful prognostic indicators, particularly with regard to *BRAF* MT.

## Highlights

• Post-treatment circulating tumor *RAS*/*BRAF* dynamics serve as useful prognostic indicators
• Reversion from mutant to wild type after the treatment links to better overall survival
• Persistent or newly acquired alterations correlate with impaired clinical outcomes

## Teaser

Health sciences; Internal medicine; Medical specialty; Medicine; Oncology

## Introduction

Rat sarcoma viral oncogene homolog (*RAS*) and v-raf murine sarcoma viral oncogene homolog B1 (*BRAF*) V600E mutations (MT) stimulate the mitogen-activated protein kinase (MAPK) pathway and promote carcinogenesis and cancer growth in multiple cancers.^1^ According to the Catalog of Somatic Mutations in Cancer and The Cancer Genome Atlas databases, which compile information on somatic MT associated with cancer, the frequency of *RAS* MT in colorectal cancer (CRC) is 33–42% for *KRAS* MT, 2–9% for *NRAS* MT, and 0% for *HRAS* MT, while the frequency of *BRAF* V600E is 5–10%.^1^^,^^2^ Patients with metastatic CRC (mCRC) and *RAS* or *BRAF* V600E MT have a poorer prognosis than those with *RAS* wild-type (WT) mCRC.^3^ The TRIBE study established FOLFOXIRI plus bevacizumab as a standard treatment for young patients with right-sided, *RAS*-mutant CRC. This regimen achieved an overall survival (OS) and progression-free survival (PFS) of 37.0 and 12.8 months in patients with *RAS/BRAF* WT mCRC, respectively. However, survival outcomes were significantly lower in those with *RAS* MT mCRC (OS and PFS: 25 and 10.9 months, respectively) and those with *BRAF* V600E MT (OS and PFS: 13.4 and 7.0 months, respectively).^4^

Anti-epidermal growth factor receptor (EGFR) antibodies constitute a class of molecular-targeted drugs used in the treatment of mCRC.^5^ EGFR-targeted antibodies exert anti-tumor effects by binding to EGFR and inhibiting signaling downstream of the receptor.^6^ However, point MTs in *RAS* (*KRAS/NRAS* exons 2, 3, and 4) reduce GTPase activity, causing the RAS protein to remain in its active state bound to GTP. This causes the mutated RAS protein to continuously transmit signals downstream, resulting in resistance to anti-EGFR antibodies, as shown in several clinical trials.^7^^,^^8^^,^^9^^,^^10^^,^^11^^,^^12^ International guidelines recommend that *RAS* and *BRAF* gene testing should be performed on patients with mCRC before consideration of anti-EGFR treatment and that anti-EGFR antibodies should only be administered to patients with *RAS* WT mCRC.^13^^,^^14^^,^^15^

Repeated tissue biopsies to confirm *RAS* and *BRAF* MT status after each line of treatment are not performed in routine clinical practice,^13^^,^^14^^,^^15^ and the persistence of the original MT status through multiple lines of therapy remains unclear.^16^ Recent advances in gene MT testing methods using circulating tumor DNA (ctDNA) in the blood have made it possible to assess MT status easily and repeatedly.^17^ Among patients with *RAS* MT mCRC, *RAS* can revert to WT following treatment in some cases; this phenomenon, known as “*NeoRAS* WT,’’ has attracted considerable attention.^18^^,^^19^ Although variations exist depending on the testing method and treatment line, studies have revealed that approximately 10% of patients with *RAS* MT mCRC convert to *RAS* WT after treatment,^20^^,^^21^ thereby opening the possibility of anti-EGFR therapy as an effective approach in later lines of treatment.^22^ However, few reports based on large-scale databases have described the relationship between changes in *RAS* and *BRAF* gene status and OS. Confirmation of prognosis based on changes in gene alterations may help optimize treatment. Therefore, in this study, we evaluated the prognostic impact of post-treatment circulating tumor *RAS* and *BRAF* mutation dynamics in patients with mCRC.

## Results

### Patient characteristics

**Figure 1:**
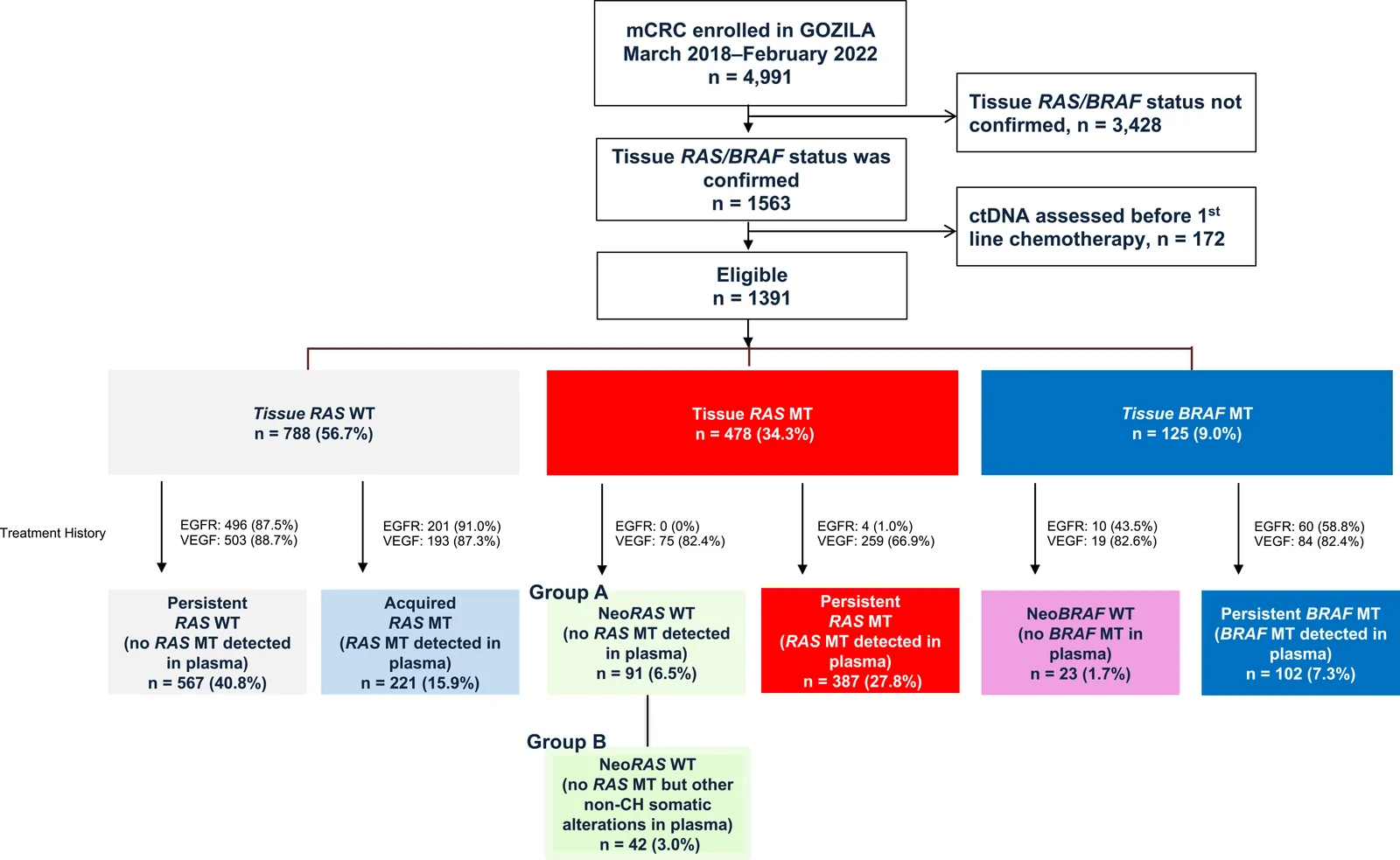
CONSORT diagram of the study ctDNA: circulating tumor DNA, *RAS*: rat sarcoma viral oncogene homolog, WT: wild-type, MT: mutant, CH: clonal hematopoiesis.

Among the 4,991 patients with mCRC enrolled in GOZILA between March 2018 and February 2022, tissue *RAS* and *BRAF* V600E MT status was confirmed in 1,563 patients before the initiation of mCRC treatment. Among these, 172 patients for whom ctDNA was measured before the administration of first-line chemotherapy were excluded, resulting in 1,391 patients whose data were evaluated. The consolidated standards of reporting trials (CONSORT) flow diagram for the study is shown in Figure 1. The median age at the time of blood sampling was 61.0 (range, 25.0–85.0) years, and 800 (57.5%) patients were male. Overall, 347 (24.9%) had right-sided tumors, and 810 (58.2%) had metastases involving multiple organs (Table 1). The liver was the most frequent site of metastasis (62.0%), followed by the lungs (47.4%), lymph nodes (32.6%), and peritoneum (26.5%). The median number of therapy lines between tissue assessment and ctDNA testing was 2 (range, 1–13). Based on baseline tissue and subsequent ctDNA testing results, patient cohorts were grouped as follows: Patients were initially classified into three cohorts: tissue *RAS/BRAF* WT (*n* = 788, 56.7%), tissue *RAS* MT (*n* = 478, 34.3%), and tissue *BRAF* MT (*n* = 125, 9.0%). Subsequent liquid biopsy (ctDNA) testing revealed dynamic shifts within these groups: persistent *RAS* WT, *n* = 567 (40.8%); persistent *RAS* MT, *n* = 387 (27.8%); acquired *RAS* MT*, n* = 221 (15.9%); Neo*RAS* WT, *n* = 91 (6.5%); persistent *BRAF* MT, *n* = 102 (7.3%); and Neo*BRAF* WT, *n* = 23 (1.7%).

**Table 1:** Patient and tumor characteristics

Characteristics	Persistent *RAS* WT (*n* = 567)	Neo*RAS* WT (*n* = 91)	Acquired *RAS* MT (*n* = 221)	Persistent *RAS* MT (*n* = 387)	Persistent *BRAF* MT (*n* = 102)	Neo*BRAF* WT (*n* = 23)
Age at enrollment, years						
Median [range]	60.0 [26.0–85.0]	60.0 [32.0–79.0]	62.0 [25.0–85.0]	62.0 [25.0–85.0]	59.0 [27.0–82.0]	63.0 [41.0–75.0]
Sex						
Male	335 (59.1)	46 (50.5)	136 (61.5)	203 (52.5)	65 (63.7)	15 (65.2)
Female	230 (40.6)	45 (49.5)	85 (38.5)	184 (47.5)	37 (36.3)	8 (34.8)
Unknown	2 (0.4)	0 (0)	0 (0.0)	0 (0)	0 (0)	0 (0)
Primary site						
Right-sided colon	112 (19.8)	27 (29.7)	42 (19.0)	126 (32.6)	35 (34.7)	6 (26.1)
Left-sided colon	451 (79.5)	64 (70.3)	178 (80.5)	261 (67.4)	66 (65.3)	17 (73.9)
Unknown	4 (0.7)	0 (0)	1 (0.5)	0 (0)	1 (1.0)	0 (0)
Cecum	20 (3.5)	8 (8.8)	7 (3.2)	50 (12.9)	14 (11.2)	3 (13.0)
Ascending colon	46 (8.1)	15 (16.5)	13 (5.9)	58 (15.0)	13 (10.4)	2 (8.7)
Transverse colon	46 (8.1)	4 (4.4)	22 (10.0)	18 (4.7)	14 (11.2)	1 (4.3)
Descending colon	38 (6.7)	4 (4.4)	11 (5.0)	14 (3.6)	13 (10.4)	2 (8.7)
Sigmoid colon	212 (37.4)	17 (18.7)	66 (29.9)	83 (21.4)	27 (21.6)	8 (34.8)
Rectum	201 (35.4)	43 (47.3)	101 (45.7)	164 (42.4)	43 (34.4)	1 (4.3)
Unknown	4 (0.7)	0 (0)	1 (0.5)	0 (0)	1 (0.8)	0 (0)
Metastatic site						
Single organ	255 (45.0)	51 (56.0)	95 (43.0)	118 (30.5)	50 (49.0)	8 (34.8)
Multi-organ	312 (55.0)	39 (42.9)	126 (57.0)	267 (69.0)	52 (51.0)	15 (65.2)
Unknown	0 (0.0)	1 (1.1)	0 (0)	2 (0.5)	0 (0)	
Liver						
Present	367 (64.7)	21 (23.1)	137 (62.0)	251 (64.9)	67 (66.3)	19 (82.6)
Absent	200 (35.3)	70 (76.9)	84 (38.0)	136 (35.1)	34 (33.7)	4 (17.4)
Lung						
Present	229 (40.4)	51 (56.0)	102 (46.2)	239 (61.8)	30 (29.4)	9 (39.1)
Absent	338 (59.6)	40 (44.0)	119 (53.8)	148 (38.2)	72 (70.6)	14 (60.9)
Lymph node						
Present	199 (35.1)	15 (16.5)	76 (34.4)	119 (30.7)	35 (34.3)	9 (39.1)
Absent	368 (64.9)	76 (83.5)	145 (65.6)	268 (69.3)	67 (65.7)	14 (60.9)
Peritoneum						
Present	136 (24.0)	38 (41.8)	62 (28.1)	95 (24.5)	32 (31.4)	5 (21.7)
Absent	431 (76.0)	53 (58.2)	159 (71.9)	292 (75.5)	70 (68.6)	18 (78.3)
Bone						
Present	45 (7.9)	2 (2.2)	14 (6.3)	35 (9.0)	5 (4.9)	2 (8.7)
Absent	522 (92.1)	89 (97.8)	207 (93.7)	352 (91.0)	97 (95.1)	21 (91.3)
Other	66 (11.6)	12 (13.2)	30 (13.6)	38 (9.8)	12 (11.8)	3 (13.0)
Treatment history						
Anti-VEGF antibody	496 (87.5)	75 (82.4)	201 (91.0)	259 (66.9)	84 (82.4)	19 (82.6)
Anti-EGFR antibody	503 (88.7)	0 (0)	193 (87.3)	4 (1.0)	60 (58.8)	10 (43.5)
Treatment lines at the time of sampling						
First	190 (33.5)	36 (39.6)	46 (20.8)	104 (26.9)	49 (48.0)	13 (56.5)
Second	115 (20.3)	28 (30.8)	44 (19.9)	107 (27.6)	24 (23.5)	4 (17.4)
Third	113 (19.9)	14 (15.4)	58 (26.2)	68 (17.6)	16 (15.7)	1 (4.3)
Fourth	63 (11.1)	6 (6.6)	38 (17.2)	56 (14.5)	6 (5.9)	4 (17.4)
Fifth	42 (7.4)	3 (3.3)	21 (9.5)	24 (6.2)	6 (5.9)	0 (0.0)
Sixth or later	44 (7.9)	4 (4.4)	35 (15.9)	28 (7.3)	7 (6.9)	0 (0.0)
First, second, and third line	418 (73.7)	78 (85.7)	148 (67.0)	279 (72.1)	89 (87.3)	19 (82.6)
Fourth line or later	149 (26.3)	13 (14.3)	73 (33.0)	108 (27.9)	13 (12.7)	4 (17.4)
cfDNA fraction						
≥1.0%	442 (78.0)	10 (11.0)	202 (91.4)	309 (79.8)	85 (83.3)	10 (43.5)
<1.0%	125 (22.0)	81 (89.0)	19 (8.6)	78 (20.2)	17 (16.7)	13 (56.5)

RAS: rat sarcoma viral oncogene homolog, BRAF: v-raf murine sarcoma viral oncogene homolog B1, WT: wild-type, MT: mutant type, VEGF: vascular endothelial growth factor, EGFR: epidermal growth factor receptor.

### Association of clinicopathological characteristics with molecular profiles

In the full Neo*RAS* WT cohort (Group A), the prevalence of liver (23.1%, *p* < 0.001), lymph node (16.5%, *p* < 0.001), and multi-organ metastasis (42.9%, *p* < 0.001) was lower than that in the other cohorts. However, lung (56.0%, *p* < 0.001) and peritoneal (41.8%, *p* = 0.004) metastases were more common than those in the other cohorts (Table 1).

Similarly, in the Neo*RAS* WT (Group B), the prevalence of liver (35.7%, *p* < 0.001), lymph node (21.4%, *p* < 0.001), and multi-organ metastasis (45.2%, *p* < 0.001) was lower; lung (61.9%, *p* < 0.001) and peritoneal (31.0%, *p* = 0.004) metastases were more common than those in other cohorts. (Table S1). Treatment with anti-EGFR antibody was most common among patients without *RAS* MT on initial tissue testing (Table 1). Notably, the frequency of sampling during first-to third-line treatment was significantly higher in patients with *BRAF* MT at initial tissue testing (Table 1). Furthermore, the proportion of patients with a maximum ctDNA variant allele frequency (VAF) of ≥1% was significantly lower in the Neo*RAS* group, followed by the Neo*BRAF* WT group (Table 1).

### Incidence of gene alteration in each cohort

The incidences of specific baseline (tissue) *RAS* MT and acquired (ctDNA) *RAS* MT are shown in Figure S2A. The most common baseline *KRAS* MT was in codons 12 and 13, detected in 71.3 and 16.3% of patients, respectively. At disease progression, *KRAS* codon 12 and 13 mutations were detected in 51.4 and 10.5% of samples, respectively. The most commonly acquired *RAS* MT was *KRAS* codon 61 (55.5%), followed by *NRAS* codon 61 (18.2%). At the time of disease progression, the maximum VAF of any *RAS* MT in patients with acquired *RAS* MT was significantly lower than in those with persistent *RAS* MT (0.79% vs. 6.9%, *p* < 0.0001; Figure S2B). Furthermore, the relative clonality of *RAS* MT in the acquired *RAS* MT cohort was significantly lower than that in the persistent *RAS* MT cohort (8.9% vs. 80.2%, *p* < 0.0001; Figure S2C). However, no significant difference was observed in terms of the proportion of patients with a maximum ctDNA VAF (any gene) of ≥1% between the acquired *RAS* MT and persistent *RAS* MT groups (Table 1). The proportion of samples with multiple *KRAS* MT detected in ctDNA was also significantly higher in patients with acquired *RAS* MT (42.7% vs. 4.1%, *p* < 0.0001).

The incidence of non-*RAS* genetic alterations in each group is presented in Table 2. The acquired *RAS* MT cohort had a significantly higher rate of genetic alterations than that in the other groups, especially in genes related to receptor tyrosine kinases (*EGFR*, *FGFR2*, and *MET*), the RAS-RAF-MAPK pathway (*MAPK*1-3), and the PI3K-AKT-mTOR pathway (*PTEN*, *mTOR*, *TSC1*, and *PDGFR*). Furthermore, the persistent *BRAF* MT cohort exhibited a significantly higher rate of genetic alterations (*BRCA1*, *SMAD4*, and *TP53*) and tended to have a higher rate of genetic alterations (*ARIDA1*, *PIK3CA,* and *MYC* amplification) than those in the Neo*BRAF* WT group.

**Table 2:** Incidence of genomic co-alterations in each group

Gene alteration	Persistent *RAS* WT (*n* = 567)	Neo*RAS* WT (*n* = 91)	Acquired *RAS* MT (*n* = 221)	Persistent *RAS* MT (*n* = 387)	Persistent *BRAF* MT (*n* = 102)	Neo*BRAF* WT (*n* = 23)
Mutations						
Receptor tyrosine kinase						
*EGFR*	112 (19.8)	6 (6.6)	85 (38.5)	44 (11.4)	60 (58.8)	10 (43.5)
*ERBB2*	54 (9.5)	3 (3.3)	18 (8.1)	24 (6.2)	7 (6.9)	0 (0)
*FGFR2*	26 (4.6)	2 (2.2)	25 (11.3)	32 (8.3)	5 (4.9)	0 (0)
*MET*	47 (8.3)	4 (4.4)	28 (12.7)	34 (8.8)	6 (5.9)	2 (8.7)
RAS-RAF-MEK pathway						
*MAP2K1*	45 (7.9)	1 (1.1)	46 (20.8)	5 (1.3)	3 (2.9)	1 (4.3)
*MAP2K2*	5 (0.9)	1 (1.1)	9 (4.1)	1 (0.3)	2 (2.0)	0 (0)
*MAPK3*	6 (1.1)	0 (0.0)	8 (3.6)	5 (1.3)	1 (1.0)	0 (0)
PI3K-AKT-mTOR pathway						
PIK3CA	81 (14.3)	2 (2.2)	48 (21.7)	98 (25.3)	22 (21.6)	2 (8.7)
PTEN	28 (4.9)	1 (1.1)	19 (8.6)	20 (5.2)	8 (7.8)	1 (4.3)
mTOR	0 (0.0)	2 (2.2)	3 (1.4)	1 (0.3)	0 (0)	0 (0)
TSC1	11 (1.9)	0 (0.0)	10 (4.5)	6 (1.6)	2 (2.0)	0 (0)
PDGFR	37 (6.5)	0 (0.0)	37 (16.7)	41 (10.6)	0 (0)	0 (0)
Other pathways						
*SMAD4*	58 (10.2)	3 (3.3)	38 (17.2)	65 (16.8)	26 (25.5)	1 (4.3)
*MYC*	18 (3.2)	0 (0.0)	16 (7.2)	16 (4.1)	5 (4.9)	2 (8.7)
*FBXW7*	20 (3.5)	0 (0.0)	19 (8.6)	38 (9.8)	7 (6.9)	0 (0)
*ARID1A*	53 (9.3)	1 (1.1)	33 (14.9)	44 (11.4)	13 (12.7)	0 (0)
*TP53*	403 (71.1)	29 (31.9)	183 (82.8)	289 (74.7)	86 (84.3)	6 (26.1)
*BRCA1*	41 (7.2)	3 (3.3)	36 (16.3)	29 (7.5)	16 (15.7)	0 (0)
Amplifications						
*EGFR*	169 (29.8)	1 (1.1)	101 (45.7)	76 (19.6)	18 (17.6)	5 (21.7)
*ERBB2*	66 (11.6)	1 (1.1)	9 (4.1)	3 (0.8)	7 (6.9)	0 (0)
*FGFR1*	64 (11.3)	1 (1.1)	40 (18.1)	21 (5.4)	5 (4.9)	0 (0)
*MET*	29 (5.1)	0 (0.0)	19 (8.6)	1 (0.3)	6 (5.9)	0 (0)
*KRAS*	32 (5.6)	0 (0.0)	22 (10.0)	10 (2.6)	2 (2.0)	1 (4.3)
*NRAS*	7 (1.2)	0 (0.0)	15 (6.8)	5 (1.3)	1 (1.0)	0 (0)
*MTOR*	14 (2.5)	2 (2.2)	16 (7.2)	12 (3.1)	5 (4.9)	0 (0)
*MYC*	38 (6.7)	1 (1.1)	24 (10.9)	17 (4.4)	13 (12.7)	0 (0)

RAS: rat sarcoma viral oncogene homolog, BRAF: v-raf murine sarcoma viral oncogene homolog B1, EGFR: epidermal growth factor receptor, ERBB2: receptor tyrosine-protein kinase erbB-2, FGFR2: fibroblast growth factor receptor 2, MAP2K: mitogen-activated protein kinase, PTEN: phosphatase and tensin homolog, mTOR: mammalian target of rapamycin, TSC1: tuberous sclerosis 1, PDGFR: platelet-derived growth factor receptor, SMAD4: SMAD family member 4, FBXW7: F box/WD repeat-containing protein 7.

### Association of *RAS* and *BRAF* profile with OS

**Figure 2:**
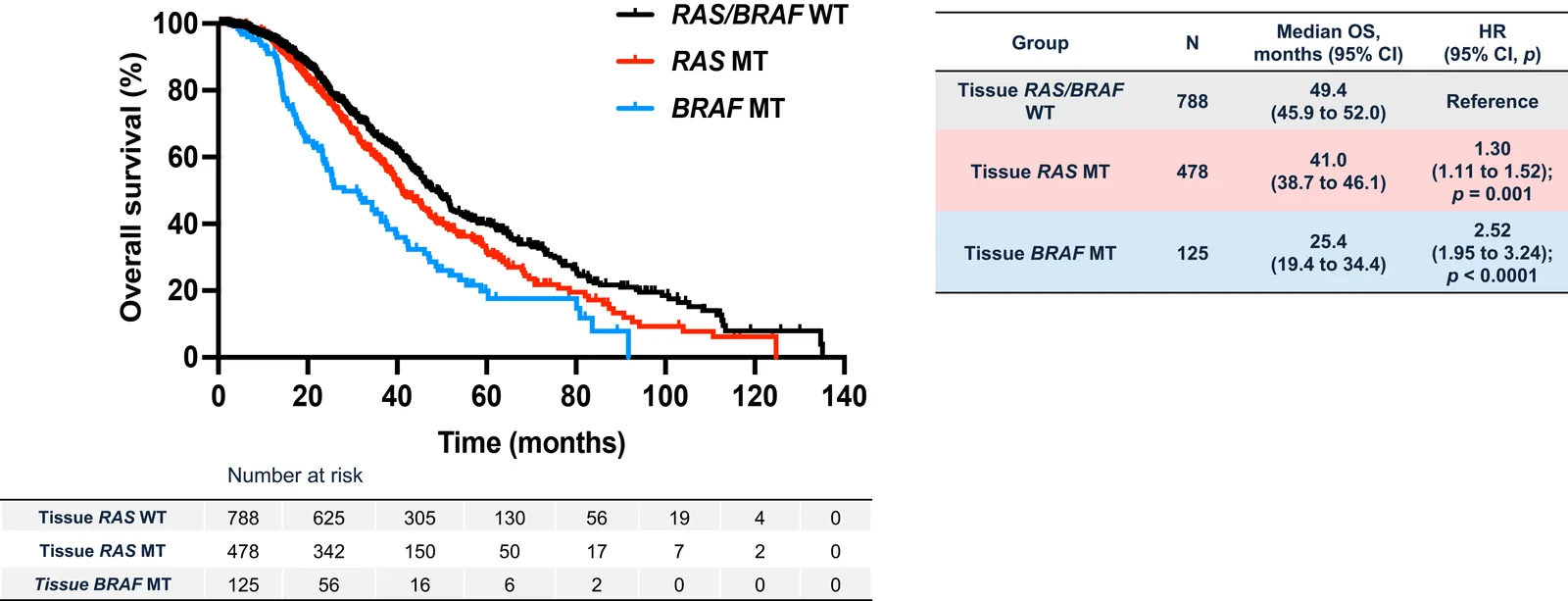
Kaplan-Meier plots of overall survival based on gene alteration (tissue *RAS* and *BRAF*) status *RAS*: rat sarcoma viral oncogene homolog, *BRAF*: Raf murine sarcoma viral oncogene homolog B, MT: mutant, WT: wild type. Statistical text for Figure 2: Data are evaluated using the Kaplan-Meier method, and survival outcomes between cohorts are compared using the two-sided log rank test. represents the number of individual patients in each cohort. The exact values are reported in the figure. Hazard ratios (HRs) and 95% confidence intervals (CIs) were calculated using univariable Cox proportional hazard regression models.

The median OS was 45.3 months (42.5–47.3 months) among all patients included in the study. Among patients stratified by tissue *RAS*/*BRAF* status, those with tissue *RAS/BRAF* WT (*n* = 788) had a median OS of 49.4 months, which served as the reference. Those with tissue *RAS* MT (*n* = 478) and *BRAF* MT (*n* = 125) were associated with a significantly shorter OS (median 41.0 and 25.4 months, respectively; hazard ratio (HR), 1.30 and 2.52, respectively; 95% CI, 1.11–1.52 *p*_*Log rank*_ = 0.001 and 1.95–3.24, *p*_*Log rank*_ < 0.0001 respectively Figure 2).

Among patients stratified by treatment exposure, those treated with anti-EGFR mAb-containing regimens had a median OS of 47.1 months, which served as the reference. Patients treated with regimens without anti-EGFR mAb had a significantly shorter OS (median 41.1 months; HR, 1.25; 95% CI, 1.08–1.45; *p*_*Log rank*_ = 0.003). Similarly, patients treated with anti-VEGF mAb-containing regimens had a median OS of 45.9 months, which served as the reference. Patients treated with regimens without anti-VEGF mAb had a significantly shorter OS (median 34.5 months; HR, 1.62; 95% CI, 1.27–2.06; *p*_*Log rank*_ < 0.0001).

Among patients stratified by the line of therapy at the time of ctDNA measurement, those treated in lines 1–3 had a median OS of 39.0 months, which served as the reference. Outperforming expectations, patients treated in line 4 or later demonstrated a significantly longer OS, with a median OS of 60.2 months (HR, 0.56; 95% CI, 0.47–0.66; *p*_*Log rank*_ < 0.0001).

**Figure 3:**
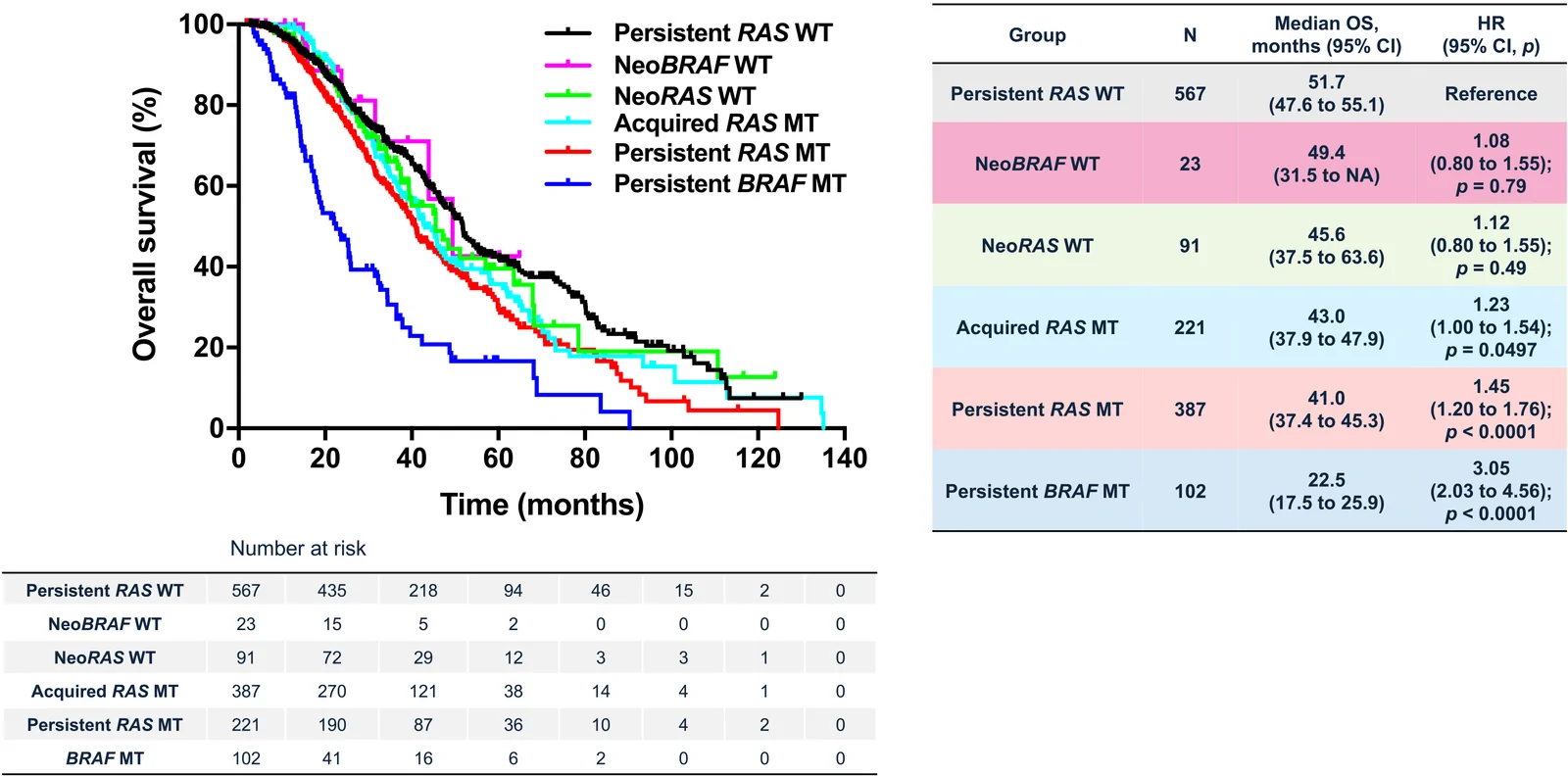
Kaplan-Meier plots of overall survival based on gene alteration (*RAS* and *BRAF* in pretreatment tissue and post treatment ctDNA) status Neo*RAS* and Neo*BRAF* cohorts were defined as those with tissue-confirmed pretreatment *RAS* and *BRAF* MTs and no post-treatment these mutations detected by ctDNA analysis. *RAS*: rat sarcoma viral oncogene homolog, MT: mutant, WT: wild-type, *BRAF*: Raf murine sarcoma viral oncogene homolog B. Statistical text for Figure 3: Data are evaluated using the Kaplan-Meier method, and survival outcomes between cohorts are compared using the two-sided log rank test. represents the number of individual patients in each cohort. The exact values are reported in the figure. Hazard ratios (HRs) and 95% confidence intervals (CIs) were calculated using univariable Cox proportional hazard regression models.

Based on post-treatment ctDNA testing results, those in the persistent *RAS* WT cohort had a median OS of 51.7 months (95% confidence interval [CI] 47.6–55.1 months), which was used as a reference for statistical comparisons. Patients with Neo*BRAF* WT had a shorter median OS, although not significant, than those with persistent *RAS* WT (49.4 months; HR, 1.08; 95% CI, 0.80–1.55; *p*_*Log rank*_ = 0.79; Figure 3); Similar outcomes were observed for Neo*RAS* WT Group A (45.6 months; HR, 1.12; 95% CI, 0.80–1.55; *p*_*Log rank*_ = 0.49; Figure 3) and Neo*RAS* WT Group B (47.1 months; HR, 1.12; 95% CI, 0.71–1.77; *p*_*Log rank*_ = 0.61; Figure S3). In contrast, OS was significantly shorter in patients in the acquired *RAS* MT cohort (median 43.0 months; HR, 1.23; 95% CI, 1.00–1.54; *p*_*Log rank*_ = 0.0497), the persistent *RAS* MT cohort (median 41.0 months; HR, 1.45; 95% CI, 1.20–1.76; *p*_*Log rank*_ < 0.0001; Figure 3), and the persistent *BRAF* MT cohort (median 22.5 months; HR, 3.05; 95% CI, 2.03–4.56; *p*_*Log rank*_ < 0.0001; Figure 3).

Compared with the other cohorts, Neo*RAS* WT Group A showed no significant difference in OS relative to the acquired *RAS* MT (HR, 0.92; 95% CI, 0.66–1.28; *p*_*Log rank*_ = 0.61) and persistent *RAS* MT (HR, 0.79; 95% CI, 0.59–1.07; *p*_*Log rank*_ = 0.13) cohorts. However, OS was significantly longer than for patients with *BRAF* MT (HR, 0.47; 95% CI, 0.32–0.67; *p*_*Log rank*_ < 0.0001). Neo*RAS* WT Group B also showed no significant difference in OS relative to the acquired *RAS* MT (HR, 0.91; 95% CI, 0.59–1.43; *p*_*Log rank*_ = 0.66) and persistent *RAS* MT (HR, 0.78; 95% CI, 0.52–1.16; *p*_*Log rank*_ = 0.19) groups. However, OS was significantly longer than that for patients with persistent *BRAF* MT (HR, 0.48; 95% CI, 0.32–0.73; *p*_*Log rank*_ = 0.0016).

Patients with Neo*BRAF* WT had a significantly longer OS than those with persistent *BRAF* MT (49.4 months vs. 22.5 months; HR, 0.31; 95% CI, 0.18–0.55; *p*_*Log rank*_ = 0.0037). In contrast, acquired *BRAF* V600E was detected in 9.5% (54/567) of patients with *RAS* WT, and these patients had a shorter median OS than those with *RAS* WT whose tumors remained *BRAF* V600 WT (46.1 vs. 51.9 months; HR, 1.46; 95% CI, 0.96–2.23; *p*_*Log rank*_ = 0.04).

To assess the robustness of our findings, a sensitivity analysis was performed using multiple ctDNA fraction cutoff values (0.5%, 1%, 2%, 5%, and 10%). All cutoffs demonstrated significant OS discrimination (HR:1.43, 1.39, 1.35, 1.47, and 1.64, respectively). A threshold of 1% was selected for subsequent analyses as it aligned with the previously reported analytical sensitivity of the Guardant360® assay (97.5% at VAF ≥1%)^23^ and showed meaningful prognostic discrimination (median OS: 51.1 months vs. 43.7 months; HR, 1.39; 95% CI, 1.17–1.64; *p* < 0.0001; Figure S4).

### Multivariable analysis results for OS

In univariable Cox proportional hazards analysis, *BRAF* or *RAS* MT in tissue prior to treatment, Neo*BRAF* WT, ctDNA fraction, treatment lines at the time of sampling, and history of anti-EGFR or anti-VEGF antibodies influenced OS. In multivariable analysis, the following factors were independently associated with shorter OS: *BRAF* MT in tissue before treatment (HR, 2.75; 95% CI, 2.10–3.61; *p* < 0.001); ctDNA fraction ≥1.0% (HR, 1.58; 95% CI, 1.31–1.91; *p* < 0.001); *RAS* MT in tissue before treatment (HR, 1.31; 95% CI, 1.00–1.70; *p* = 0.049); and treatment lines at the time of sampling (HR, 1.77; 95% CI, 1.50–2.10; *p* < 0.001). History of anti-VEGF antibodies (0.70, 95% CI, 0.55–0.90, *p* < 0.001) and Neo*BRAF* WT (HR, 0.37; 95% CI, 0.16–0.85; *p* = 0.019) were associated with longer OS (Table 3).

**Table 3:** Cox proportional hazard analysis for overall survival

Overall survival	Univariable analysis				Multivariable analysis			
	HR	Lower 95% CI	Upper 95% CI	*p* value	HR	Lower 95% CI	Upper 95% CI	*p* value
Sex (Female∗ or Male)	0.9	0.78	1.05	0.21				
Age (<65∗ or ≥65 years)	0.95	0.61	1.5	0.84				
Primary tumor location (left∗ or right)	1.12	0.94	1.32	0.2				
Liver metastasis (negative∗ or positive)	1.04	0.90	1.21	0.59				
Lung metastasis (negative∗ or positive)	0.99	0.86	1.16	0.99				
Lymph node metastases (negative∗ or positive)	0.86	0.73	1.01	0.059				
Peritoneal metastasis (negative∗ or positive)	0.91	0.77	1.08	0.28				
Multiple organ metastases (negative∗ or positive)	0.96	0.83	1.12	0.63				
*RAS* mutation in tissue prior to therapy initiation (negative∗ or positive)	1.17	1.006	1.37	0.042	1.31	1	1.70	0.049
Acquired *RAS* mutation (negative∗ or positive)	1.07	0.87	1.32	0.51				
*Neo RAS* WT (negative∗ or positive)	0.78	0.56	1.08	0.13				
*BRAF* V600E mutation in tissue prior to therapy initiation (negative∗ or positive)	2.28	1.79	2.92	<0.001	2.75	2.1	3.61	<0.001
*Neo BRAF* WT (negative∗ or positive)	0.31	0.13	0.72	0.006	0.37	0.16	0.85	0.019
Treatment history of anti-VEGF antibody (no∗ or yes)	0.61	0.48	0.79	<0.001	0.7	0.55	0.9	<0.001
Treatment history of anti-EGFR antibody (no∗ or yes)	0.8	0.69	0.93	0.0028	0.92	0.72	1.19	0.53
Treatment lines at the time of sampling (1st, 2nd, and 3rd∗ or 4th or later)	1.8	1.52	2.12	<0.001	1.77	1.5	2.1	<0.001
ctDNA fraction (<1.0%∗, ≥1.0%)	1.39	1.15	1.67	<0.001	1.58	1.31	1.91	<0.001

RAS: rat sarcoma viral oncogene homolog, BRAF: v-raf murine sarcoma viral oncogene homolog B1, WT: wild-type, MT: mutant type, HR: hazard ratio, CI: confidence interval, VEGF: vascular endothelial growth factor, EGFR: epidermal growth factor receptor, ∗Reference.

## Discussion

To the best of our knowledge, this is the largest study to evaluate the relationship between OS and changes in *RAS* and *BRAF* status using ctDNA in patients with mCRC. In this study, the median OS associated with Neo*RAS* and *NeoBRAF* WT mCRC was shorter than but not significantly different from that of patients with *RAS* WT. Other *RAS* and *BRAF* mutation cohorts had significantly shorter OS than did patients with *RAS* WT. Furthermore, patients with Neo*BRAF* WT had a significantly longer median OS than those with persistent *BRAF* MT. Moreover, non-*RAS* gene alterations were more frequent in the acquired *RAS* MT cohort, especially for genes related to the RAS-RAF-MAPK and PI3K-AKT-mTOR pathways.

The absence of detectable ctDNA after treatment is associated with a longer OS.^24^^,^^25^^,^^26^ One of the reasons for this is that the ctDNA VAF correlates with tumor volume,^27^ and patients with a low tumor volume may have a good treatment response and prognosis. In our study, low ctDNA VAF after initial therapy was indeed associated with better OS in the multivariable analysis. This may reflect relative tumor sensitivity before chemotherapy or a less aggressive, slower-growing phenotype. To better understand whether ctDNA-negative cases explained the lack of statistical difference between the Neo*RAS* WT and *RAS* WT cohorts, we compared OS for Neo*RAS* WT Group B, which included only cases with ctDNA evidence of non-*RAS* alterations, to the full *RAS* WT cohort and found similar results. Therefore, the absence of *RAS* MT in ctDNA after chemotherapy may be associated with improved survival in mCRC. The tumors of patients in this group may have a similar biology to those of patients with *RAS* WT, raising the hypothesis that tumors in this group may share biological features with *RAS* WT mCRC, though whether this translates into anti-EGFR responsiveness remains to be prospectively confirmed. As previously reported, an anti-EGFR antibody was administered to six patients in this cohort and was effective in three patients.^22^ Several clinical trials are ongoing in this setting,^28^ and the OS may potentially improve if ongoing clinical trials confirm the efficacy of anti-EGFR antibodies in this setting; however, this remains speculative pending the availability of definitive clinical evidence.

Loss of *BRAF* V600E after chemotherapy may also contribute to improved survival. Among patients with *BRAF* MT detected by tumor tissue testing at baseline, 18.4% had no *BRAF* MT in ctDNA after treatment, a phenomenon known as Neo*BRAF* WT mCRC.^29^ Regarding the relationship between post-treatment ctDNA status and treatment prognosis, biomarker analysis in the FIRE 4.5 study showed that treatment response and outcomes were better in patients with decreased *BRAF* MT VAF after treatment and in those who did not have *BRAF* MT in post-treatment ctDNA.^30^ Furthermore, some patients with *BRAF* MT displayed a good prognosis, in contrast to the traditionally poor prognosis.^31^ Therefore, the confirmation of *BRAF* MT status in post-treatment ctDNA may also help estimate prognosis and optimize the treatment strategy in *BRAF* MT mCRC.

Patients with persistent *RAS* MT had shorter OS than those with *RAS* WT. *RAS* mutations are poor prognostic factors for patients with CRC and poor predictors of response to anti-EGFR antibodies.^32^^,^^33^ In addition, patients with acquired *RAS* MT had shorter OS than those with *RAS* WT. The main reason for this is that acquired *RAS* MT is among the factors causing resistance to anti-EGFR antibodies.^34^ Moreover, the rate of non-*RAS* alterations was significantly higher in the acquired *RAS* MT group than in the other groups. In particular, alterations related to the RAS-RAF-MEK, PI3K-AKT-mTOR, and other pathways such as myelocytomatosis oncogene (*MYC*) were significantly higher in this group. These are often reported to cause resistance to both the primary and acquired alterations associated with anti-EGFR antibodies.^35^ In cases where these alterations occur, various pathways are activated, and extremely complex resistance mechanisms are thought to lead to reduced OS. Furthermore, acquired *RAS* MT also reportedly affects the therapeutic efficacy of re-challenge with anti-EGFR antibodies in later-line treatment.

In a sub-analysis of an anti-EGFR antibody re-challenge study, Cremolini et al. reported that treatment efficacy was lower in patients in whom ctDNA *RAS* was detected before anti-EGFR antibody re-challenge.^36^ Furthermore, even when ctDNA was restricted to *RAS* WT before anti-EGFR antibody re-challenge, such as in the REMMARY and PERSUIT trials, which examined the efficacy and safety of anti-EGFR antibody re-challenge, no cases were reported where anti-EGFR antibodies were effective under conditions where new *RAS* MT appeared even transiently.^37^ To improve the outcomes of patients with acquired resistance, using molecular-targeted drugs in combination with chemotherapy and avoiding their use alone is preferable.^38^ This is likely because, when chemotherapy is not used in combination, acquired resistance can develop more easily, even when treatment lines differ. This is likely a major reason why cytotoxic chemotherapy has been selected in several ongoing clinical trials of molecular-targeted drugs for *BRAF*, *HER2,* and *KRAS* MT mCRC. Another approach is to use MT-specific inhibitors that target acquired resistance MTs; preclinical studies have shown that this has a therapeutic effect.^35^ However, as few studies have targeted subclonal MTs in clinical settings,^39^ further research is expected.

### Conclusion

Post-treatment circulating tumor *RAS* and *BRAF* MT dynamics may serve as useful prognostic indicators, particularly with regard to *BRAF* MT.

### Limitations of the study

Our study has some limitations. First, the retrospective design introduces inherent selection bias and limits causal inference, as data collection and patient selection were not prospectively controlled; furthermore, the validity and reproducibility of diagnosis could be improved in cases with low VAF, particularly those with <1%VAF.^40^ Second, the cohort exhibited considerable heterogeneity with respect to prior treatment history, tumor characteristics, and ctDNA sampling timing, which may have confounded the prognostic associations observed. In addition, immortal-time bias was present, as patients with poor prognosis were not included in this study; to mitigate this, analyses were restricted to patients who underwent post-treatment ctDNA assessment, though residual bias cannot be excluded.^41^ Therefore, OS was extended beyond the previously reported period. Third, ctDNA was not measured serially before and after each treatment cycle; thus, whether ctDNA and tissue *RAS* status matched was not confirmed. The absence of serial paired tissue-plasma sampling further limits mechanistic interpretation and prevents definitive conclusions regarding clonal evolution under therapeutic pressure. Finally, the lack of independent external validation represents a critical limitation; these findings should therefore be considered exploratory and require prospective confirmation in larger, independent cohorts before recommending clinical application. Despite these limitations, our results suggest that confirming gene alterations in ctDNA after treatment may be useful for estimating prognosis and optimizing treatment selection in mCRC.

## Resource availability

### Lead contact

Requests for further information and resources should be directed to and will be fulfilled by the lead contact, Eiji Shinozaki (eiji.shinozaki@jfcr.or.jp).

### Materials availability

This study did not generate new unique reagents.

### Data and code availability

#### Data

De-identified individual patient-level clinical variables and circulating tumor DNA profiling data reported in this study are available from the lead contact upon reasonable request, subject to institutional data sharing compliance.

#### Code

This study did not report original computer code, algorithms, or computational models. Standard commercially available and open-source packages (R version 4.2.1, GraphPad Prism 7, and EZR version 1.61) were utilized as outlined in the key resources table.

#### Additional information

Any additional information required to reanalyze the data reported in this paper is available from the lead contact upon request.

## Acknowledgments

This work was supported by the National Cancer Center Research and Development Fund (31-A-5 to A. Ohtsu) and SCRUM-Japan Funds (http://www.scrum-japan.ncc.go.jp/index.html). The authors would like to thank the patients and their families, investigators, research nurses, and study coordinators who participated in this study. Collaborators in the GI-SCREEN and GOZILA Study included: Yukiya Narita and Toshiki Masuishi (Aichi Cancer Center Hospital), Kazuhiro Yoshida (Gifu University Hospital), Akihito Tsuji (Kagawa University Hospital), Yuta Adachi and Koushiro Ohtsubo (10.13039/100018502Kanazawa University), Junji Furuse (10.13039/100030735Kyorin University), Eiji Oki (10.13039/501100004096Kyushu University), Ken Kato (National Cancer Center Hospital), Yosuke Horita (10.13039/501100009337Saitama Medical University), Akiyoshi Kanazawa (Shimane Prefectural Central Hospital), Kentaro Yamazaki (10.13039/100019728Shizuoka Cancer Center), Takako Nakajima (St. Marianna University), and Toshikazu Moriwaki (University of Tsukuba Hospital).

## Author contributions

H.O.: conceptualization lead; data curation lead; formal analysis lead; investigation lead; methodology lead; project administration lead; writing—original draft lead; E.S.: conceptualization equal; project administration lead; supervision lead; writing—review and editing lead; Y.N.: funding acquisition lead; methodology lead; resources lead; visualization lead; writing—review and editing lead; T.Y.: funding acquisition lead; methodology lead; project administration lead; resources lead; supervision equal; writing—review and editing supporting; writing—review and editing supporting; T.E., H.T., H.S., Y.S., Y.Ko, Y.Ka, T.D., M.S., T.S., T.N., M.G., N.T., T.K., H.B.

## Declaration of interests

Eiji Shinozaki received honoraria from Takeda Pharma, Merck, Lily, and Chugai Pharma.

Yoshiaki Nakamura received honoraria from Chugai Pharma, Guardant Health AMEA, and Merck and research funding from Taiho Pharmaceutical (Inst), Guardant Health (Inst), Genomedia (Inst), Chugai Pharma (Inst), Guardant Health (Inst), Seattle Genetics (Inst), and Roche (Inst).

Taito Esaki received honoraria from Lilly, Taiho Pharmaceutical, Daiichi Sankyo, and Chugai Pharma and research funding from Daiichi Sankyo (Inst), MSD (Inst), Novartis (Inst), Ono Pharmaceutical (Inst), Astellas Pharma (Inst), Lilly (Inst), Bayer (Inst), Nihon Kayaku (Inst), Amgen Astellas BioPharma (Inst), Parexel (Inst), IQVIA (Inst), Quintiles (Inst), Eisai (Inst), Pfizer (Inst), Chugai Pharma (Inst), Syneos Health (Inst), Asahi Kasei Pharma (Inst), Amgen (Inst), Dainippon Sumitomo (Inst), and Dainippon Sumitomo (Inst).

Hisateru Yasui received honoraria from Taiho Pharmaceutical, Chugai Pharma, Bristol Myers Squibb Japan, Daiichi Sankyo, Lilly, Yakult Honsha, Bayer Yakuhin, Takeda, and Ono Pharmaceutical and research funding from MSD (Inst), Ono Pharmaceutical (Inst), Astellas Pharma (Inst), AstraZeneca (Inst), and Daiichi Sankyo (Inst).

Hiroya Taniguchi received honoraria from Bayer, Sanofi, Takeda, Chugai Pharma, Taiho Pharmaceutical, Lilly, Merck Serono, Yakult Honsha, Medical & Biological Laboratories Co., Ltd, Bristol Myers Squibb Japan, MSD K.K., Novartis, Daiichi Sankyo, Mitsubishi Tanabe Pharma, Nippon Kayaku, and Ono Yakuhin and research funding from Dainippon Sumitomo Pharma (Inst), Array BioPharma (Inst), MSD Oncology (Inst), Ono Pharmaceutical (Inst), Daiichi Sankyo (Inst), Sysmex (Inst), Novartis (Inst), and Takeda (Inst).

Satake Hironaga received honoraria from Bristol Myers Squibb Co., Ltd., Bayer Co., Ltd., Chugai Pharmaceutical Co., Ltd, Daiichi Sankyo Co., Ltd., Eli Lilly Japan Co., Ltd., Merck Bio Pharma Co., Ltd., MSD Co., Ltd., Ono Pharmaceutical Co., Ltd., Sanofi Co., Ltd., Taiho Pharmaceutical Co., Ltd., Takeda Co., Ltd. and Yakult Honsha Co., Ltd. and research funding from Ono Pharmaceutical Co Ltd, Daiichi Sankyo, Taiho Pharmaceutical Co Ltd, and Takeda Pharmaceutical Co., Ltd.

Yu Sunakawa received honoraria from Taiho Pharmaceutical, Chugai Pharma, Takeda, Bayer Yakuhin, Bristol Myers Squibb Japan, Lilly, Merck, Sysmex, MSD K.K., Ono Pharmaceutical, Daiichi Sankyo, Guardant Health, and Incyte, has a consulting/advisory role at Bristol Myers Squibb Japan, MSD K.K., Daiichi Sankyo, and Merck, and received research funding from Taiho Pharmaceutical, Takeda, Chugai Pharma, Lilly, Sanofi, and Otsuka.

Yoshito Komatsu received honoraria from Lilly Japan, Taiho Pharmaceutical, Chugai Pharma, Takeda, Bayer Yakuhin, Bristol Myers Squibb Co, Sanofi/Aventis, Merck, Yakult Honsha, Ono Pharmaceutical, Nipro Corporation, Moroo Co, Asahi Kasei, Mitsubishi Tanabe Pharma, Otsuka, Medical Review Co., Ltd, and Daiichi Sankyo and research funding from MSD K.K., Taiho Pharmaceutical, Yakult Honsha, Bayer Yakuhin, DAIICHI SANKYO CO., Ltd, Ono Pharmaceutical, NanoCarrier, Eisai, Sanofi/Aventis, Sysmex, Shionogi, IQVIA, Parexel International Corporation, Astellas Pharma, Mediscience Planning, Sumitomo Dainippon Pharma Co., Ltd, A2 Healthcare, Incyte, Lilly (Inst), Nipro Corporation (Inst), and BeiGene (Inst).

Yoshinori Kagawa has a consulting/advisory role at Taiho Pharmaceutical, Merck, and Lilly, received speakers’ bureau honoraria from Lilly, Sanofi, Takeda, Merck, Taiho Pharmaceutical, MSD, Chugai Pharma, Yakult Pharmaceutical, Bayer, and Ono Pharmaceutical, and received research funding from Ono Pharmaceutical.

Tadamichi Denda received honoraria from Daiichi Sankyo, Ono Pharmaceutical, and Sysmex and research funding from Ono Pharmaceutical (Inst), Amgen (Inst), MSD, Pfizer, and Bristol Myers Squibb Foundation.

Shiozawa Manabu received honoraria from Lilly, Takeda, Taiho, Ono, Yakult, and Merck.

Taroh Satoh received honoraria: Chugai Pharma, Merck Serono, Bristol Myers Squibb, Takeda, Yakult Honsha, Lilly, Bayer Yakuhin, Ono Pharmaceutical, Merck, Astellas Pharma, Taiho Pharmaceutical, Nihon Kayaku, and Daiichi Sankyo, has a consulting/advisory role at Bayer Yakuhin, Lilly, Ono Pharmaceutical, Takara Bio, Merck Serono, and Nihon Kayaku, and received research funding from Yakult Honsha (Inst), Chugai Pharma (Inst), Ono Pharmaceutical (Inst), Sanofi (Inst), Lilly (Inst), Daiichi Sankyo (Inst), Merck (Inst), Merck Serono (Inst), Gilead Sciences (Inst), Dainippon Sumitomo Pharma (Inst), and IQVIA (Inst).

Tomohiro Nishina received honoraria from Taiho Pharmaceutical, Ono Pharmaceutical, Bristol Myers Squibb Japan, Daiichi Sankyo, and Chugai Pharma and research funding from MSD (Inst), Ono Pharmaceutical (Inst), Astellas Pharma (Inst), Daiichi Sankyo/UCB Japan (Inst), Bristol Myers Squibb Japan (Inst), Chugai Pharma (Inst), Taiho Pharmaceutical (Inst), and AstraZeneca (Inst).

Toshihumi Yamaguchi received honoraria from Daiichi Sankyo Company, Limited, Ono Pharmaceutical, Taiho Pharmaceutical, MSD K.K, Takeda, Sumitomo Dainippon Pharma Co., Ltd, Yakult Pharmaceutical, and Lilly and research funding from Chugai Pharma, Taiho Pharmaceutical, and Nippon Kayaku.

Naoki Takahashi received honoraria from Ono Pharmaceutical, Bristol Myers Squibb Japan, and Taiho Pharmaceutical.

Takeshi Kato received honoraria from Chugai Pharma, Ono Pharmaceutical, Takeda, Lilly, and Asahi Kasei and research funding from Chugai Pharma.

Hideaki Bando received honoraria from Taiho, Lilly, and Ono. M.W. Nihon Medi-Physics and a research grant from Ono Pharmaceutical.

Kensei Yamaguchi has a consulting/advisory role at Bristol Myers Squibb Japan and Daiichi Sankyo, received honoraria for a speakers’ bureau from Chugai Pharma, Bristol Myers Squibb Japan, Takeda, Taiho Pharmaceutical, Lilly, Ono Pharmaceutical, Daiichi Sankyo, and Merck, and received research funding from Ono Pharmaceutical (Inst), Taiho Pharmaceutical (Inst), Daiichi Sankyo (Inst), Lilly (Inst), Gilead Sciences (Inst), Yakult Honsha (Inst), Chugai Pharma (Inst), Boehringer Ingelheim (Inst), Eisai (Inst), MSD Oncology (Inst), Sanofi (Inst), and Bristol Myers Squibb (Inst).

Takayuki Yoshino received honoraria from Chugai Pharma, Takeda Pharma, Merck, Ono Pharmaceutical, and MSD K.K, consulting fee from Sumitomo Corp, and research funding from MSD (Inst), Daiichi Sankyo Company, Limited (Inst), Ono Pharmaceutical (Inst), Taiho Pharmaceutical (Inst), Amgen (Inst), Sanofi (Inst), Pfizer (Inst), Sysmex (Inst), Nippon Boehringer Ingelheim (Inst), Chugai Pharma (Inst), isai (Inst), Bristol Myers Squibb (Inst), Caris MPI (Inst), Exact Sciences (Inst), FALCO biosystems (Inst), Medical & Biological Laboratories (Inst), Merus (Inst), Miyarisan Pharmaceutical (Inst), Molecular Health (Inst), Natera (Inst), Roche Diagnostics (Inst), and Takeda Pharmaceutical (Inst).

## Declaration of generative AI and AI-assisted technologies in the writing process

During the preparation of this work, the authors used Notebook LM to assist in checking grammar. After using this tool/service, the authors reviewed and edited the content as needed and take full responsibility for the content of the publication.

## STAR★Methods

### Key resources table

**Table:** 

REAGENT or RESOURCE	SOURCE	IDENTIFIER
**Critical commercial assays**		

Guardant360® (74-gene panel assay)	Guardant Health, Inc.	https://www.guardanthealth.com/; RRID:SCR_023455
RASKET-B KIT	Medical & Biological Laboratories (MBL) Co., Ltd.	Cat# 4505; https://www.mblbio.com/

**Deposited data**		

De-identified clinical and ctDNA alteration profiles	This study	Available from the lead contact upon reasonable request

**Software and algorithms**		

R version 4.2.1	R Project for Statistical Computing	https://www.r-project.org/; RRID:SCR_001905
GraphPad Prism 7	GraphPad Software	https://www.graphpad.com/; RRID:SCR_002798
EZR (Easy R) version 1.61	Saitama Medical Center, Jichi Medical University	https://www.jichi.ac.jp/saitama-sct/SaitamaHP.files/statmedEN.html

**Other**		

UMIN Clinical Trials Registry (UMIN-CTR)	UMIN-CTR	Trial registration number: UMIN000029315; https://center6.umin.ac.jp/cgi-open-bin/ctr_e/ctr_view.cgi?recptno=R000033509

### Experimental model and study participant details

#### Patients

This retrospective, multi-institutional study evaluated a cohort of patients with metastatic colorectal cancer (mCRC) enrolled in the nationwide plasma genomic profiling platform, SCRUM-Japan GOZILA, across 31 core cancer institutions in Japan. All data collection and clinical protocols were approved by the Institutional Review Board of the Cancer Institute Hospital of the Japanese Foundation for Cancer Research (approval number: 2021-GB-009). The study was conducted in strict compliance with the ethical principles mandated by the Declaration of Helsinki. The protocol was described on the hospital’s website, and participants were given the opportunity to opt out of the study. Data from next-generation sequencing (NGS; Guardant360®, Guardant Health, Inc., Redwood City, CA, USA) were used to confirm the subsequent ctDNA genotypes (*RAS*, *BRAF*, and others) following chemotherapy. Patients with mCRC enrolled in the GOZILA study between March 2018 and February 2022, with confirmed results from tissue *RAS* and *BRAF* testing before chemotherapy initiation, were eligible, regardless of the treatment line, as shown in Figure S1. The GOZILA study is a nationwide plasma genomic profiling study involving 31 core cancer institutions in Japan. Patients with metastatic gastrointestinal cancer were eligible for enrollment.

A total of 1,391 patients who underwent baseline tissue sequencing and subsequent post-treatment ctDNA next-generation sequencing were eligible for final survival analysis. All participating subjects in this cohort were Japanese ethnicity. The median age of the entire cohort at the time of plasma sampling was 61.0 years (range, 25.0–85.0 years). In terms of biological sex distribution, the cohort comprised 800 male patients (57.5%) and 591 female patients (42.5%), with sex data missing or unknown for 2 patients (0.1%).

To rigorously assess the potential confounding influence of baseline demographic characteristics on clinical outcomes, both biological sex and age were integrated into the univariable Cox proportional hazard models for OS. Univariable analysis demonstrated that neither biological sex (Male vs. Female: HR, 0.90; 95%CI, 0.78–1.05; *p* = 0.21) nor age group (<65 vs. ≥65 years: HR, 0.95; 95% CI, 0.61–1.50; *p* = 0.84) exerted a statistically significant impact or interaction on survival outcomes. Consequently, these demographic variables were not identified as independent prognostic indicators and were excluded from the final multivariable Cox regression models.

### Method details

#### Blood samples, ctDNA isolation and sequencing

NGS of ctDNA was performed using the Guardant360® system (Guardant Health, Inc., Redwood City, CA, USA), a liquid biopsy platform leveraging a 74-gene panel. This assay was configured to detect single-nucleotide variants, insertions/deletions, fusions, and copy number alterations across the complete coding regions of target oncogenes, including *KRAS*, *NRAS*, and *BRAF*, alongside the analytical determination of microsatellite instability status. For sample acquisition in the SCRUM-Japan GOZILA study, 2 × 10 mL of whole blood was collected from each participant into cell-free DNA BCT® tubes (Streck, Inc., La Vista, NE, USA) containing proprietary cellular stabilization reagents to prevent the lysis of nucleated cells and subsequent genomic DNA contamination.^23^^,^^24^ The average sequencing depth was 15,000×, and the ctDNA fraction was determined from the maximum variant allelic fraction. Blood specimens were maintained at ambient temperature and shipped to a centralized laboratory at Guardant Health for processing within standard pre-analytical stability windows. Cell-free DNA (cfDNA) was isolated from plasma via automated magnetic bead-based extraction protocols. Following extraction, cfDNA underwent oligonucleotide hybridization capture and targeted library preparation utilizing unique molecular identifiers (UMIs) to convert individual double-stranded DNA fragments into digital sequences, thereby suppressing polymerase chain reaction (PCR) errors and sequencing artifacts. Sequencing was executed on high-throughput NGS platforms (Illumina, Inc., San Diego, CA, USA) with a target average sequencing depth of coverage per base. The definitive ctDNA fraction for each clinical specimen was quantitatively determined from the maximum variant allele frequency (VAF) observed among the detected somatic alterations.

#### Tumor tissue DNA sequencing

All enrolled patients were histopathologically diagnosed with metastatic colorectal cancer (mCRC) through microscopic evaluation of tissue specimens obtained via diagnostic biopsy or surgical resection. Prior to the initiation of first-line treatment for advanced disease, routine clinical *RAS* (*KRAS* and *NRAS*) and *BRAF* mutational profiling of the primary or metastatic tumor tissue was mandated using validated standard-of-care assays. As the primary representative measurement method utilized across cohorts, the RASKET-B KIT (Medical & Biological Laboratories [MBL] Co., Ltd., Nagoya, Japan) was applied using genomic DNA extracted from formalin-fixed paraffin-embedded (FFPE) tumor tissue sections in absolute accordance with the manufacturer's operational protocol. The assay applies a multiplex PCR-based reverse sequence-specific oligonucleotide (PCR-rSSO) methodology combined with xMAP® technology on the Luminex platform, allowing for high-throughput, multiplexed fluorometric detection of target alleles within a single reaction well. Through specific biotinylated primer amplification and hybridization to sequence-specific oligonucleotide probes conjugated to distinct fluorescent microbeads, we rigorously evaluated 12 discrete types of *RAS* exon 2 mutations (G12S, G12C, G12R, G12D, G12V, G12A, G13S, G13C, G13R, G13D, G13V, and G13A), 8 types of *RAS* exon 3 mutations (A59T, A59G, Q61K, Q61E, Q61L, Q61P, Q61R, and Q61H), 4 types of *RAS* exon 4 mutations (K117N, A146T, A146P, and A146V), and the *BRAF* exon 15 V600E hotspot mutation. Fluorescent signals were swept and analyzed on a Luminex analyzer to determine positive mutational status based on predefined institutional cut-off bead intensities.

#### Endpoints and data collection

The primary endpoint was to compare OS among cohorts of patients with mCRC according to changes in their tumor genomic profiles from baseline tissue to subsequent ctDNA testing. Patients were divided into six groups: persistent *RAS* WT, persistent *RAS* MT, change from *RAS* MT to *RAS* WT (Neo*RAS* WT), change from *RAS* WT to *RAS* MT (acquired *RAS* MT), persistent *BRAF* V600E (*BRAF* MT), and change from *RAS* MT to *RAS* WT (Neo*BRAF* WT). *BRAF* MT other than V600E was not considered. Furthermore, patients with Neo*RAS* WT were categorized into two groups based on genetic alterations: Group A, tissue-confirmed pretreatment *RAS* MT and no post-treatment *RAS* MT detected by ctDNA analysis; however, Group A included both patients with true biological clearance of *RAS* mutations and undetectable ctDNA levels as patients with insufficient tumor-derived DNA shedding would fulfill the Group A criteria irrespective of their underlying *RAS* mutational status. To address this limitation, Group B was defined more stringently to include patients with tissue-confirmed pretreatment *RAS* MT and undetectable post-treatment *RAS* MT, but with other post-treatment ctDNA alterations remaining after excluding those potentially associated with clonal hematopoiesis (CH), thereby confirming the presence of ctDNA and providing greater confidence that *RAS* mutation absence reflects genuine clonal elimination rather than assay insensitivity. Mutations in *TP53, HRAS, PDGFRA*, and *GNAS* genes were considered potential CH alterations.^42^^,^^43^ Secondary endpoints included the differences in clinicopathological factors among the five groups. Data on the following features were collected: patient age and sex, primary tumor location, metastatic site, treatment lines at the time of sampling, and history of anti-EGFR or anti-VEGF antibodies. OS was measured from the time of first-line treatment initiation to the date of death.

### Quantification and statistical analysis

Clinicopathological factors and distribution frequencies were compared between the Persisitent*RAS* WT cohort (reference group) and other molecular subgroups. Continuous variables were evaluated using the Mann–Whitney U test, and categorical variables were analyzed using the Chi-square test or Fisher's exact test as appropriate. Overall survival (OS) was calculated from the date of first-line therapy initiation to the date of death. Survival curves were estimated using the Kaplan–Meier method, and statistical differences between the genomic dynamics cohorts were evaluated using the two-sided log-rank test. The exact number of individual patients for each cohort is detailed in Table 1 and the respective figure legends. To identify independent prognostic factors, variables achieving a *p*-value < 0.05 in the univariable analysis were subsequently entered into the multivariable Cox proportional hazard regression models. Key statistical values, including hazard ratios (HRs), 95% confidence intervals (CIs), and exact values, are explicitly reported in the text, figures, and tables. A two-sided value *p* < 0.05 was considered statistically significant for all analyses. All statistical calculations were performed using the “EZR” software (Saitama Medical Center, Jichi Medical University, Saitama, Japan), which is a graphical user interface for R (The R Foundation for Statistical Computing, Vienna, Austria, version 4.2.1) and R Commander.^44^
