# Supplementary material for: Prognostic impact of circulating tumor RAS and BRAF mutation dynamics in patients with metastatic colorectal cancer
Source: iScience. 2026 Aug 13;29(9):117112. doi: 10.1016/j.isci.2026.117112 (PMC13499150; doi:10.1016/j.isci.2026.117112)
Supplement: Supplementary file 1 [file mmc1.pdf]

**iScience, Volume *29***

## Supplemental information

# Prognostic impact of circulating tumor *RAS* and *BRAF* mutation dynamics in patients with metastatic colorectal cancer

**Hiroki Osumi, Eiji Shinozaki, Yoshiaki Nakamura, Taito Esaki, Hisateru Yasui, Hiroya Taniguchi, Hironaga Satake, Yu Sunakawa, Yoshito Komatsu, Yoshinori Kagawa, Tadamichi Denda, Manabu Shiozawa, Taroh Satoh, Tomohiro Nishina, Toshifumi Yamaguchi, Naoki Takahashi, Takeshi Kato, Hideaki Bando, Kensei Yamaguchi, and Takayuki Yoshino**

Figure S1

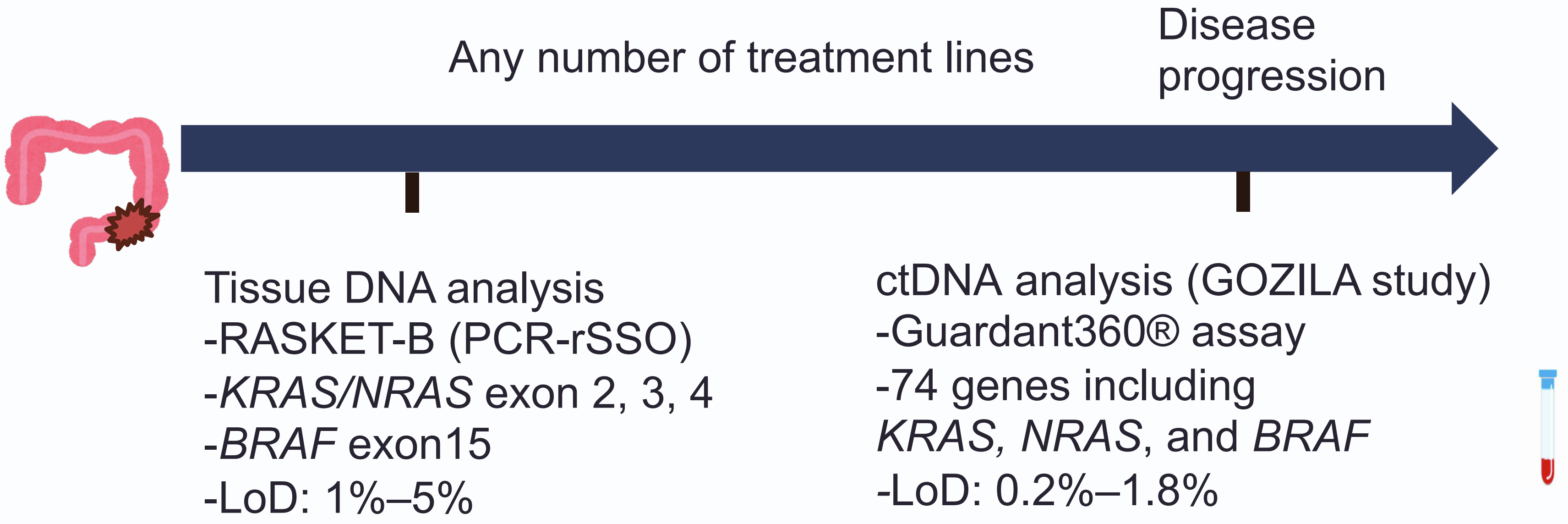
Any number of treatment lines
Disease progression
Tissue DNA analysis
-RASKET-B (PCR-rSSO)
-*KRAS/NRAS* exon 2, 3, 4
-*BRAF* exon15
-LoD: 1%–5%
ctDNA analysis (GOZILA study)
-Guardant360® assay
-74 genes including
*KRAS, NRAS*, and *BRAF*
-LoD: 0.2%–1.8%

**Fig. S2**

a)

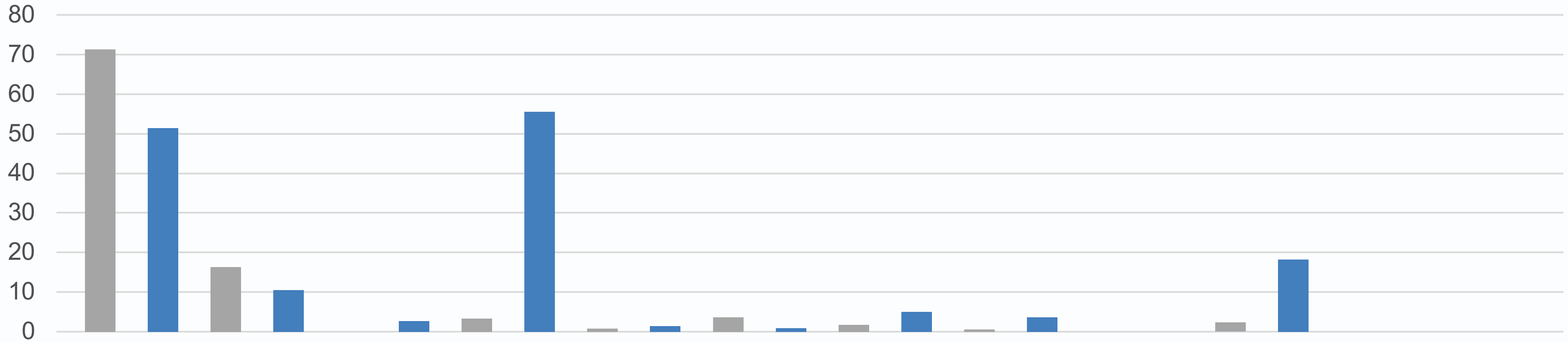


| | *KRAS* exon2 | | *KRAS* exon3 | | *KRAS* exon4 | | *NRAS* exon2 | | *NRAS* exon3 | | *NRAS* exon4 | |
|---|---|---|---|---|---|---|---|---|---|---|---|---|
| Codon | 12 | 13 | 59 | 61 | 117 | 146 | 12 | 13 | 59 | 61 | 117 | 146 |
| *RAS* MT (Original MT detected in tissue ) (%) | 71.3 | 16.3 | 0 | 3.3 | 0.8 | 3.6 | 1.7 | 0.6 | 0 | 2.3 | 0 | 0 |
| Acquired *RAS MT* (%) | 51.4 | 10.5 | 2.7 | 55.5 | 1.4 | 0.9 | 5.0 | 3.6 | 0 | 18.2 | 0 | 0 |

b)

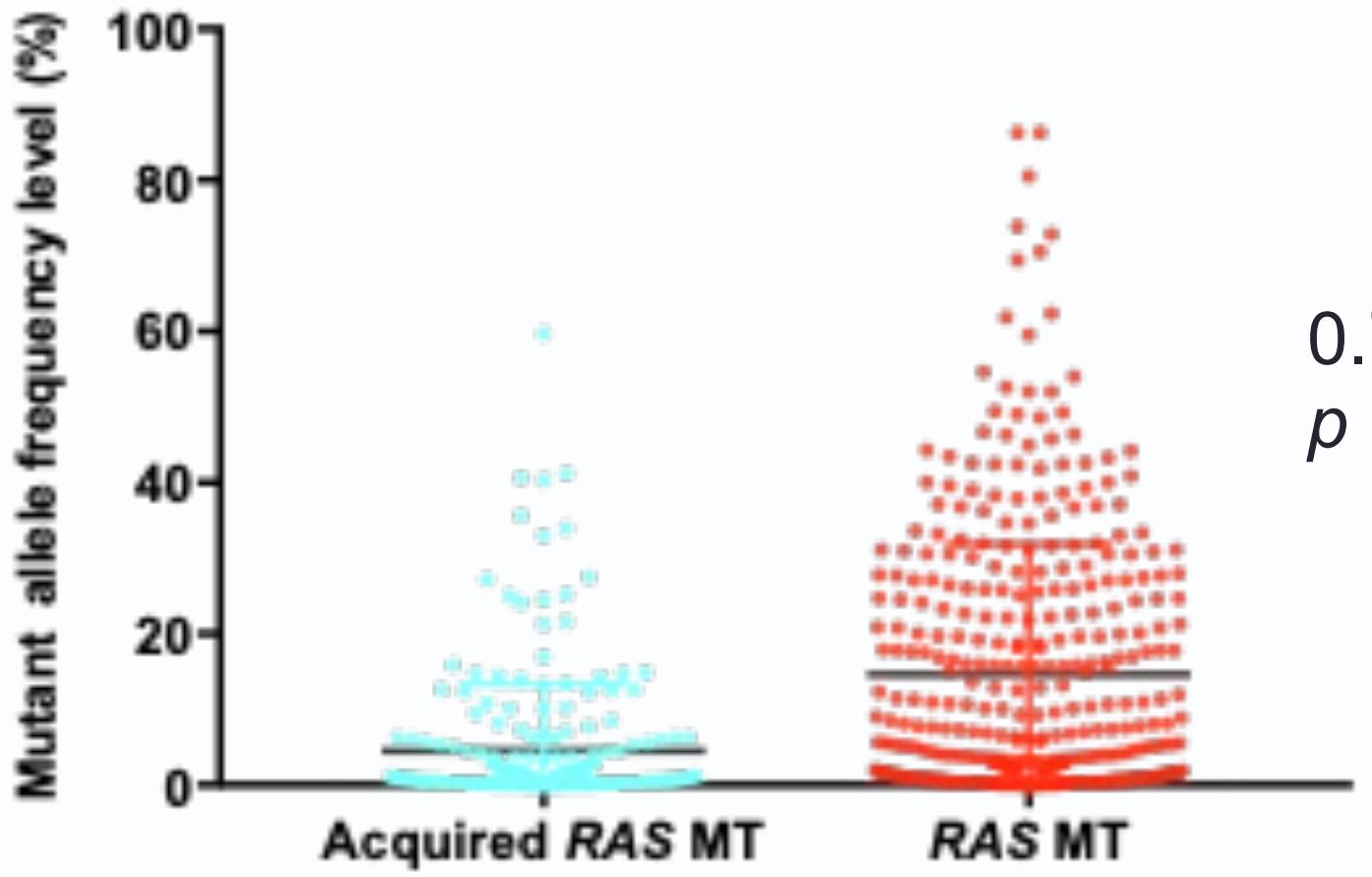


0.79% vs. 6.9%, $p < 0.001$

c)

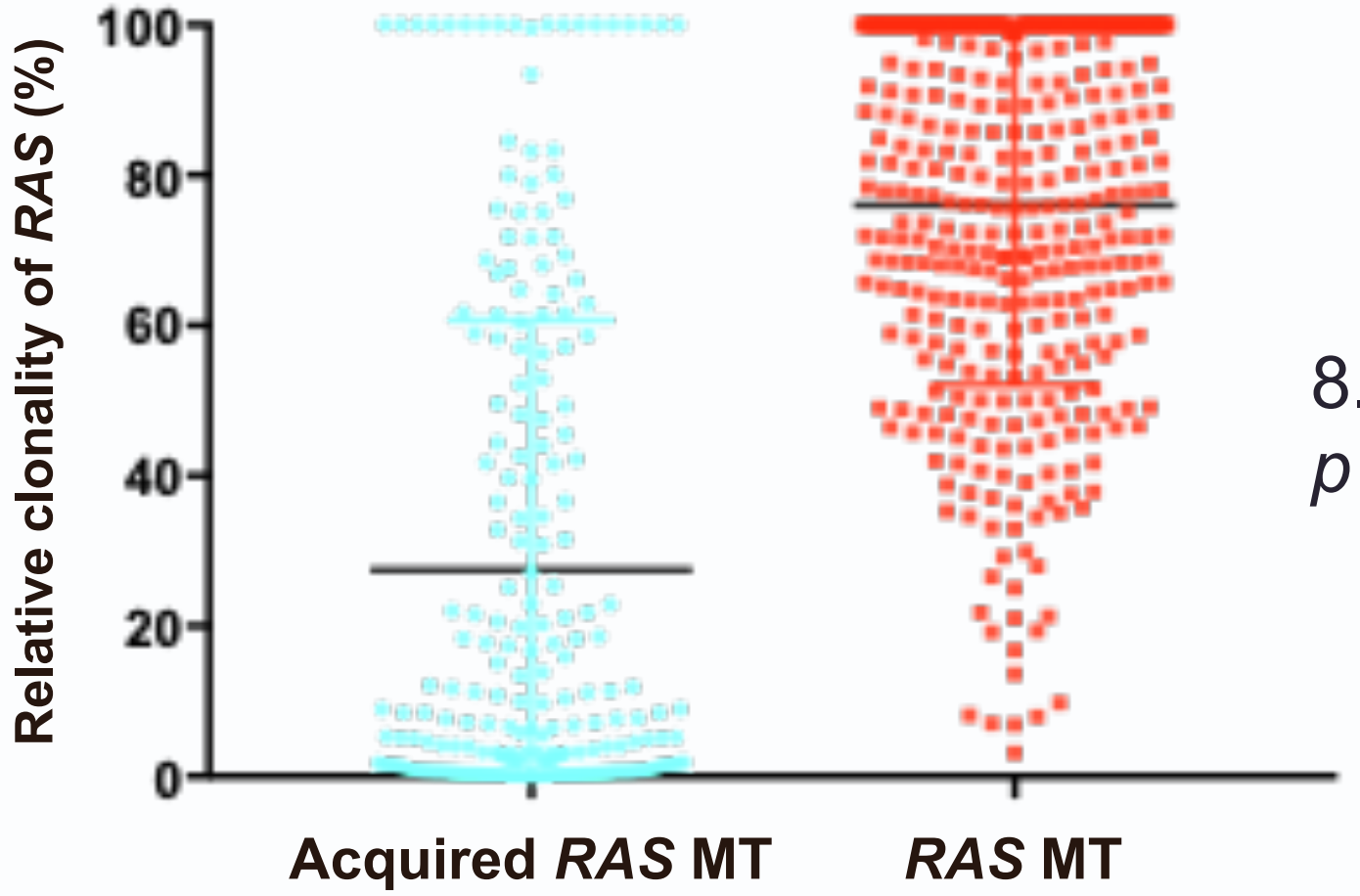


8.9% vs. 80.2%, $p < 0.001$

## Figure S3

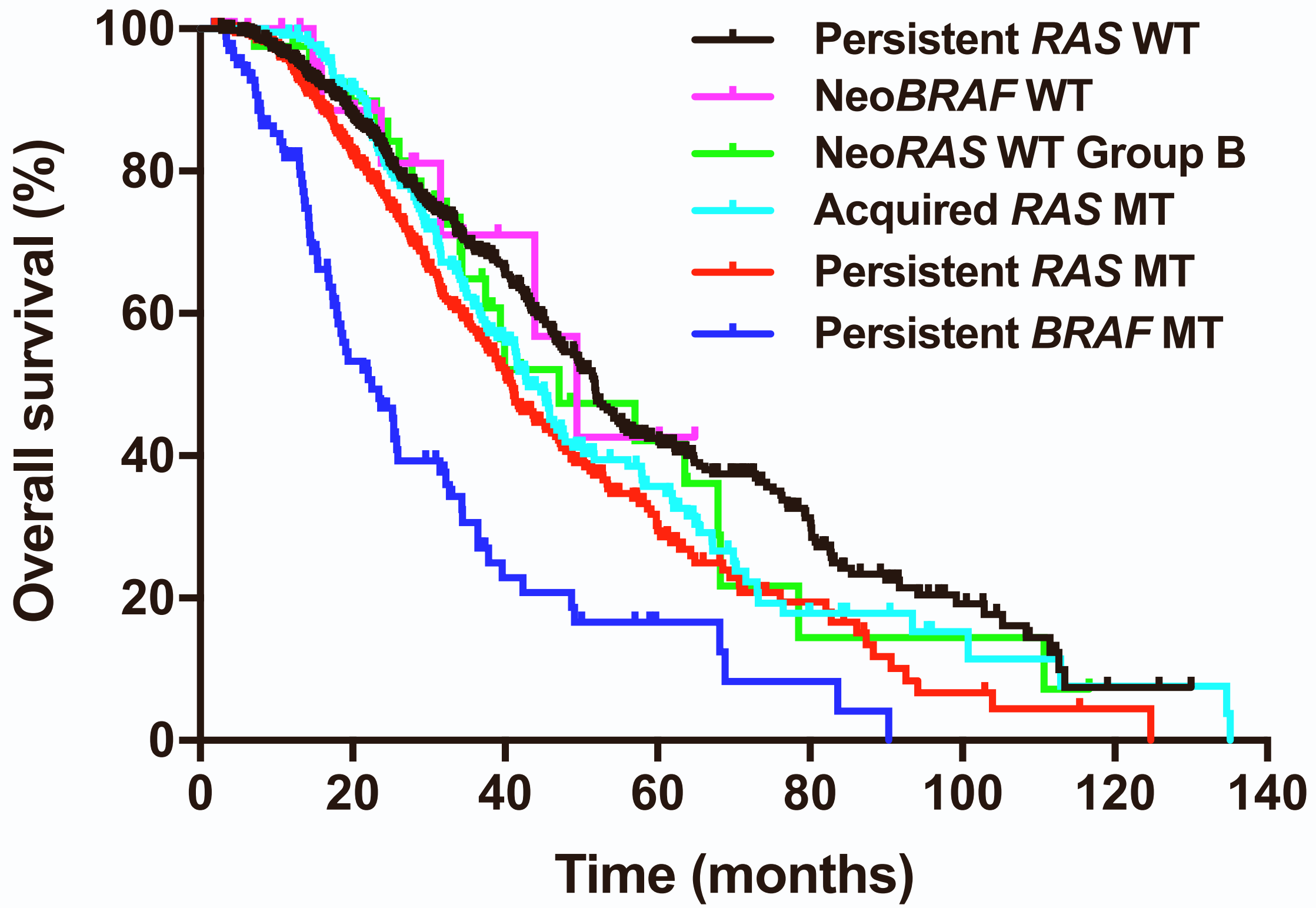


| Group | N | Median OS, months (95% CI) | HR (95% CI, p ) |
|---|---|---|---|
| Persistent *RAS* WT | 567 | 51.7 (47.6 to 55.1) | Reference |
| Neo*BRAF* WT | 23 | 49.4 (31.5 to NA) | 1.08 (0.80 to 1.55); p = 0.79 |
| Neo*RAS* WT Group B | 42 | 47.1 (37.5 to 63.6) | 1.12 (0.71 to 1.77); p = 0.61 |
| Acquired *RAS* MT | 221 | 43.0 (37.9 to 47.9) | 1.23 (1.00 to 1.54); p = 0.0497 |
| Persistent *RAS* MT | 387 | 41.0 (37.4 to 45.3) | 1.45 (1.20 to 1.76); p < 0.0001 |
| Persistent *BRAF* MT | 102 | 22.5 (17.5 to 25.9) | 3.05 (2.03 to 4.56); p < 0.0001 |

Number at risk

| | | | | | | | | |
|---|---|---|---|---|---|---|---|---|
| Persistent *RAS* WT | 567 | 435 | 218 | 94 | 46 | 15 | 2 | 0 |
| Neo*BRAF* WT | 23 | 15 | 5 | 2 | 0 | 0 | 0 | 0 |
| Neo*RAS* WT GroupB | 42 | 37 | 13 | 9 | 3 | 3 | 0 | 0 |
| Acquired *RAS* MT | 387 | 270 | 121 | 38 | 14 | 4 | 1 | 0 |
| Persistent *RAS* MT | 221 | 190 | 87 | 36 | 10 | 4 | 2 | 0 |
| *BRAF* MT | 102 | 41 | 16 | 6 | 2 | 0 | 0 | 0 |

## Figure S4

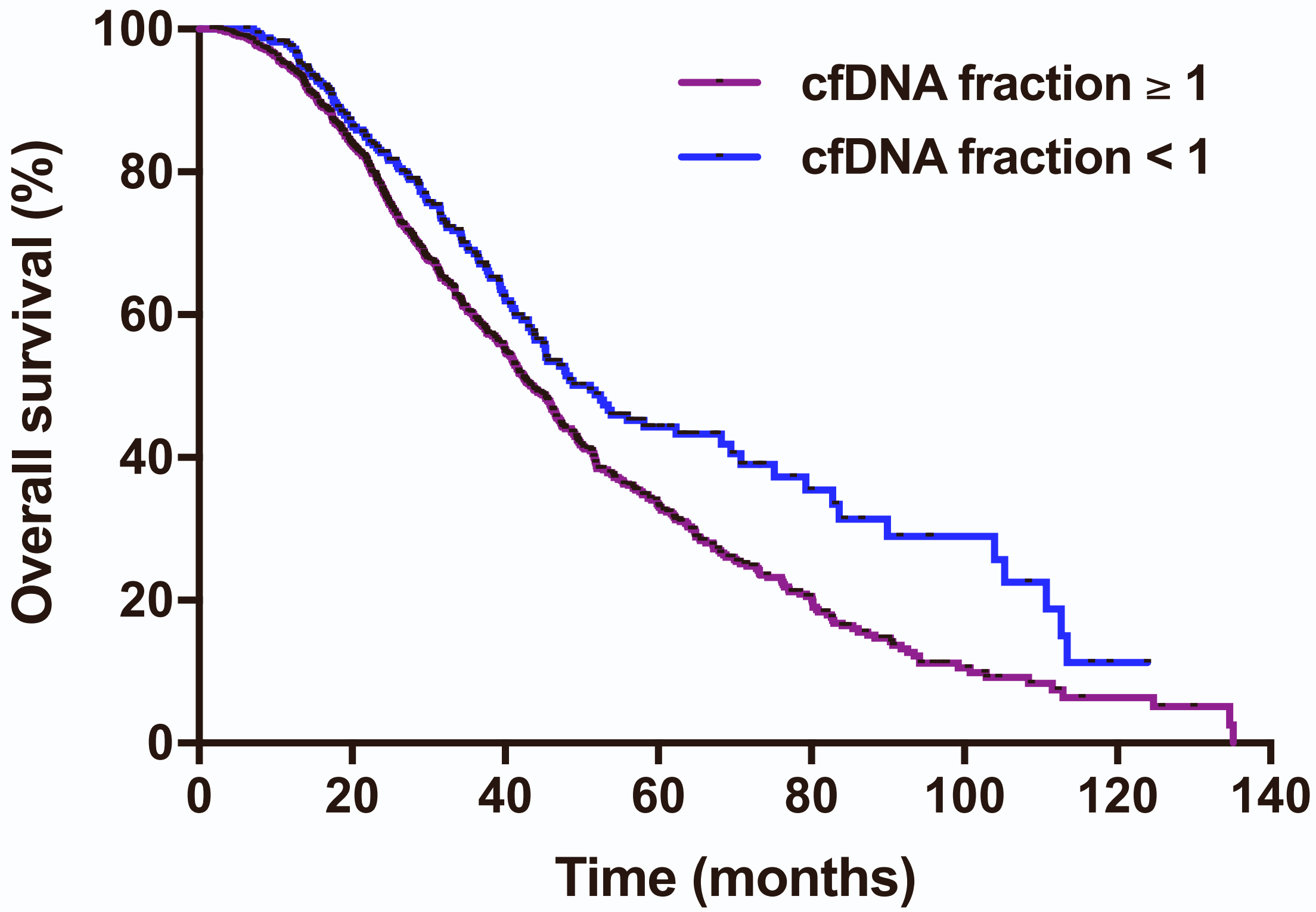


| Group | N | Median OS, months (95% CI) | HR (95% CI, p ) |
|---|---|---|---|
| cfDNA fraction <1 | 333 | 51.1 (43.9 to 68.3) | Reference |
| cfDNA fraction ≥ 1 | 1058 | 43.7 (41.1 to 46.6) | 1.39 (1.17 to 1.64); $p < 0.0001$ |

Number at risk

| | | | | | | | | |
|---|---|---|---|---|---|---|---|---|
| cfDNA fraction <1 | 333 | 249 | 118 | 47 | 18 | 9 | 1 | 0 |
| cfDNA fraction ≥ 1 | 1058 | 774 | 353 | 139 | 57 | 17 | 5 | 0 |

**Table S1. Patient and tumor characteristics**

| Characteristics | Persistent *RAS* WT (n = 567) | Neo*RAS* WT Group B (n = 42) | Acquired *RAS* MT (n = 221) | Persistent *RAS* MT (n = 387) | *BRAF* MT (n = 102) | *NeoBRAF* MT (n = 23) |
|---|---|---|---|---|---|---|
| **Age at enrollment, years** | | | | | | |
| Median [range] | 60.0 [26.0–85.0] | 62.5 [33.0–78.0] | 62.0 [25.0–85.0] | 62.0 [25.0–85.0] | 59.0 [27.0–82.0] | 63.0 [41.0–75.0] |
| **Sex** | | | | | | |
| Male | 335 (59.1) | 20 (47.6) | 136 (61.5) | 203 (52.5) | 65 (63.7) | 15 (65.2) |
| Female | 230 (40.6) | 22 (52.4) | 85 (38.5) | 184 (47.5) | 37 (36.3) | 8 (34.8) |
| Unknown | 2 ( 0.4) | 0 (0) | 0 ( 0.0) | 0 (0) | 0 (0) | 0 (0) |
| **Primary site** | | | | | | |
| Right-sided colon | 112 (19.8) | 13 (31.0) | 42 (19.0) | 126 (32.6) | 35 (34.7) | 6 (26.1) |
| Left-sided colon | 451 (79.5) | 29 (69.0) | 178 (80.5) | 261 (67.4) | 66 (65.3) | 17 (73.9) |
| Unknown | 4 ( 0.7) | 0 (0) | 1 ( 0.5) | 0 (0) | 1 (1.0) | 0 (0) |
| Cecum | 20 ( 3.5) | 2 (4.8) | 7 ( 3.2) | 50 (12.9) | 14 (11.2) | 3 (13.0) |
| Ascending colon | 46 ( 8.1) | 9 (21.4) | 13 ( 5.9) | 58 (15.0) | 13 (10.4) | 2 ( 8.7) |
| Transverse colon | 46 ( 8.1) | 2 (4.8) | 22 (10.0) | 18 (4.7) | 14 (11.2) | 1 ( 4.3) |
| Descending colon | 38 ( 6.7) | 0 (0) | 11 ( 5.0) | 14 (3.6) | 13 (10.4) | 2 ( 8.7) |
| Sigmoid colon | 212 (37.4) | 10 (23.8) | 66 (29.9) | 83 (21.4) | 27 (21.6) | 8 (34.8) |
| Rectum | 201 (35.4) | 19 (45.2) | 101 (45.7) | 164 (42.4) | 43 (34.4) | 1 ( 4.3) |
| Unknown | 4 ( 0.7) | 0 (0) | 1 ( 0.5) | 0 (0) | 1 ( 0.8) | 0 (0) |
| **Metastatic site** | | | | | | |
| Single organ | 255 (45.0) | 22 (52.4) | 95 (43.0) | 118 (30.5) | 50 (49.0) | 8 (34.8) |
| Multi-organ | 312 (55.0) | 19 (45.2) | 126 (57.0) | 267 (69.0) | 52 (51.0) | 15 (65.2) |
| Unknown | 0 ( 0.0) | 1 (2.4) | 0 (0) | 2 (0.5) | 0 (0) | |
| Liver | | | | | | |
| Present | 367 (64.7) | 15 (35.7) | 137 (62.0) | 251 (64.9) | 67 (66.3) | 19 (82.6) |
| Absent | 200 (35.3) | 27 (64.3) | 84 (38.0) | 136 (35.1) | 34 (33.7) | 4 (17.4) |
| Lung | | | | | | |
| Present | 229 (40.4) | 26 (61.9) | 102 (46.2) | 239 (61.8) | 30 ( 29.4) | 9 (39.1) |
| Absent | 338 (59.6) | 16 (38.1) | 119 (53.8) | 148 (38.2) | 72 ( 70.6) | 14 (60.9) |
| Lymph node | | | | | | |
| Present | 199 (35.1) | 9 (21.4) | 76 (34.4) | 119 (30.7) | 35 ( 34.3) | 9 (39.1) |
| Absent | 368 (64.9) | 33 (78.6) | 145 (65.6) | 268 (69.3) | 67 ( 65.7) | 14 (60.9) |
| Peritoneum | | | | | | |
| Present | 136 (24.0) | 13 (31.0) | 62 (28.1) | 95 (24.5) | 32 ( 31.4) | 5 (21.7) |
| Absent | 431 (76.0) | 29 (69.0) | 159 (71.9) | 292 (75.5) | 70 ( 68.6) | 18 (78.3) |
| Bone | | | | | | |
| Present | 45 ( 7.9) | 2 (4.8) | 14 ( 6.3) | 35 (9.0) | 5 (4.9) | 2 (8.7) |
| Absent | 522 (92.1) | 40 (95.2) | 207 (93.7) | 352 (91.0) | 97 (95.1) | 21 (91.3) |
| Other | 66 (11.6) | 0 (0) | 30 (13.6) | 38 (9.8) | 12 (11.8) | 3 (13.0) |
| **Treatment history** | | | | | | |
| Anti-VEGF antibody | 496 (87.5) | 35 (83.3) | 201 (91.0) | 259 (66.9) | 84 (82.4) | 19 (82.6) |
| Anti-EGFR antibody | 503 (88.7) | 0 (0) | 193 (87.3) | 4 (1.0) | 60 (58.8) | 10 (43.5) |
| **Treatment lines at the time of sampling** | | | | | | |
| First | 190 (33.5) | 18 (42.9) | 46 (20.8) | 104 (26.9) | 49 (48.0) | 13 (56.5) |
| Second | 115 (20.3) | 10 (23.8) | 44 (19.9) | 107 (27.6) | 24 (23.5) | 4 (17.4) |
| Third | 113 (19.9) | 6 (14.3) | 58 (26.2) | 68 (17.6) | 16 (15.7) | 1 ( 4.3) |
| Fourth | 63 (11.1) | 5 (11.9) | 38 (17.2) | 56 (14.5) | 6 ( 5.9) | 4 (17.4) |
| Fifth | 42 ( 7.4) | 1 (2.4) | 21 ( 9.5) | 24 (6.2) | 6 ( 5.9) | 0 ( 0.0) |
| Sixth or later | 44 (7.9) | 2 (4.8) | 35 (15.9) | 28 (7.3) | 7 (6.9) | 0 ( 0.0) |
| First, second, and third line | 418 (73.7) | 34 (81.0) | 148 (67.0) | 279 (72.1) | 89 (87.3) | 19 (82.6) |
| Fourth line or later | 149 (26.3) | 8 (19.0) | 73 (33.0) | 108 (27.9) | 13 (12.7) | 4 (17.4) |
| **cfDNA fraction** | | | | | | |
| ≥1.0% | 442 (78.0) | 8 (19.0) | 202 (91.4) | 309 (79.8) | 85 (83.3) | 10 (43.5) |
| <1.0% | 125 (22.0) | 34 (81.0) | 19 ( 8.6) | 78 (20.2) | 17 (16.7) | 13 (56.5) |

*RAS*: rat sarcoma viral oncogene homolog
*BRAF*: v-raf murine sarcoma viral oncogene homolog B1
WT: wild-type
MT: mutant type
VEGF: vascular endothelial growth factor
EGFR: epidermal growth factor receptor
ctDNA: circulating tumor DNA

**Figure S1.** Study schema.pptx.

ctDNA: circulating tumor DNA, *RAS*: rat sarcoma viral oncogene homolog, WT: wild-type, MT: mutant, CH: clonal hematopoiesis, mCRC: metastatic colorectal cancer.

**Figure S2.** related to Table1.

a) The incidences of specific baseline (tissue) *RAS* MT and acquired (ctDNA) *RAS* MT.pptx.

b) Comparison of the maximum VAF of any *RAS* MT between patients with acquired *RAS* MT and persistent *RAS* MT.pptx.

c) Comparison of the relative clonality of *RAS* MT between patients with acquired *RAS* MT and persistent *RAS* MT.pptx.

**Figure S3.** related to Figure 3.

Kaplan–Meier plots of overall survival based on gene alteration (*RAS* and *BRAF*) status.pptx.

Patients classified as Neo*RAS* WT were defined as having tissue-confirmed pretreatment *RAS* MTs, no post-treatment *RAS* MTs detected by ctDNA analysis, and other gene MTs confirmed post-treatment without evidence of clonal hematopoiesis. *RAS*: rat sarcoma viral oncogene homolog, MT: mutant, WT: wild-type, ctDNA: circulating tumor DNA.

**Statistical text for Figure S3:** Data are evaluated using the Kaplan–Meier method, and survival outcomes between cohorts are compared using the two-sided log-rank test. represents the number of individual patients in each cohort. The exact values are reported in the figure. Hazard ratios (HRs) and 95% confidence intervals (CIs) were calculated using univariable Cox proportional hazard regression models.

**Figure S4.** related to Table3.

Kaplan–Meier plots of overall survival based on cfDNA fraction (<1%, ≥1%). pptx.

**Statistical text for Figure S4:** Data are evaluated using the Kaplan–Meier method, and survival outcomes between cohorts are compared using the two-sided log-rank test. represents the number of individual patients in each cohort. The exact values are reported in the figure. Hazard ratios (HRs) and 95% confidence intervals (CIs) were calculated using univariable Cox proportional hazard regression models.

**Table S1, related to Table 1.**

Patient and tumor characteristics in the Neo*RAS* WT (Group B)
